# Ergocalciferol and Microcirculatory Function in Chronic Kidney Disease and Concomitant Vitamin D Deficiency: An Exploratory, Double Blind, Randomised Controlled Trial

**DOI:** 10.1371/journal.pone.0099461

**Published:** 2014-07-09

**Authors:** Gavin Dreyer, Arthur T. Tucker, Steven M. Harwood, Rupert M. Pearse, Martin J. Raftery, Muhammad M. Yaqoob

**Affiliations:** 1 Department of Renal Medicine, Barts Health NHS Trust, London, United Kingdom; 2 Department of Critical Care Medicine, Barts Health NHS Trust, London, United Kingdom; 3 Department of Translational Medicine and Therapeutics, Queen Mary University of London, London, United Kingdom; 4 Ernest Cooke Microvascular Unit, Barts Health NHS Trust, London, United Kingdom; University of Milan, Italy

## Abstract

**Background and Objectives:**

Vitamin D deficiency and endothelial dysfunction are non-traditional risk factors for cardiovascular events in chronic kidney disease. Previous studies in chronic kidney disease have failed to demonstrate a beneficial effect of vitamin D on arterial stiffness, left ventricular mass and inflammation but none have assessed the effect of vitamin D on microcirculatory endothelial function.

**Study Design:**

We conducted a randomised controlled trial of 38 patients with non diabetic chronic kidney disease stage 3–4 and concomitant vitamin D deficiency (<16 ng/dl) who received oral ergocalciferol (50,000 IU weekly for one month followed by 50,000 IU monthly) or placebo over 6 months. The primary outcome was change in microcirculatory function measured by laser Doppler flowmetry after iontophoresis of acetylcholine. Secondary endpoints were tissue advanced glycation end products, sublingual functional capillary density and flow index as well as macrovascular parameters. Parallel *in vitro* experiments were conducted to determine the effect of ergocalciferol on cultured human endothelial cells.

**Results:**

Twenty patients received ergocalciferol and 18 patients received placebo. After 6 months, there was a significant improvement in the ergocalciferol group in both endothelium dependent microcirculatory vasodilatation after iontophoresis of acetylcholine (p = 0.03) and a reduction in tissue advanced glycation end products (p = 0.03). There were no changes in sublingual microcirculatory parameters. Pulse pressure (p = 0.01) but not aortic pulse wave velocity was reduced. There were no significant changes in bone mineral parameters, blood pressure or left ventricular mass index suggesting that ergocalciferol improved endothelial function independently of these parameters. In parallel experiments, expression of endothelial nitric oxide synthase and activity were increased in human endothelial cells in a dose dependent manner.

**Conclusions:**

Ergocalciferol improved microcirculatory endothelial function in patients with chronic kidney disease and concomitant vitamin D deficiency. This process may be mediated through enhanced expression and activity of endothelial nitric oxide synthase.

**Trial Registration:**

Clinical trials.gov NCT00882401

## Introduction

Patients with chronic kidney disease (CKD) are far more likely to die of cardiovascular disease (CVD) than progress to end stage kidney disease (ESKD) [1]. Traditional risk factors including age, hypertension, smoking and diabetes mellitus do not entirely account for the excess of CVD mortality in patients with CKD. Vitamin D deficiency, a non-traditional CVD risk factor in patients with all stages of kidney disease [2], [3], [4], is highly prevalent in patients with CKD [5], [6] and is associated with elevated cardiovascular (CV) morbidity and mortality [7], [8], [9].

Vitamin D deficiency in patients with CKD has been shown to correlate with impairment in endothelial function [10], [11]. In pre-clinical [12] and clinical studies [13], endothelial dysfunction has been identified as a non-traditional risk factor for CVD in CKD with improvements in endothelial function reflecting improved global vascular health and a reduced risk of CVD [14].

Observational studies [15], [16], [17], [18], [19], [20] have provided support for the protective role of vitamin D therapy in reducing the risk of CVD in patients with CKD and ESKD. However, these studies were heterogeneous in design, therapeutic intervention and patient populations (CKD vs ESKD) and have not elucidated the mechanism by which vitamin D reduces CV risk in this patient group. Several *in vitro*, pre-clinical and clinical studies have demonstrated that endothelial and therefore microcirculatory function can be ameliorated after treatment with both activated and nutritional forms of vitamin D [21], [22], [23], [24], [25]. However, none of these studies have included patients with CKD. Two previous studies in patients with non-dialysis and CKD and ESKD evaluating the effect of nutritional vitamin D compounds on endothelial biomarkers and conduit artery function have provided conflicting results [26], [27].

The microcirculation, defined as blood vessels <150 µm in diameter [28] located within tissue parenchyma, is intricately linked to endothelial function and predicts the function of the microcirculatory beds in renal and cardiac tissue [29], [30], [31], [32], [33]. A review of cardiovascular assessment in patients with CKD [34] has highlighted the need for further assessments of microcirculatory dysfunction in patients with CKD as a method for predicting adverse CV outcomes. To date, there have been no prospective, randomized controlled studies investigating the effect of vitamin D on microcirculatory endothelial function or CV endpoints in patients with CKD [35].

We therefore conducted an exploratory, double blind, randomised, controlled trial to determine if therapy with ergocalciferol compared to placebo improves microcirculatory endothelial function in patients with CKD and concomitant vitamin D deficiency. We also conducted parallel *in vitro* experiments to elucidate the mechanistic pathway of ergocalciferol in cultured human endothelial cells.

## Methods

### Study design

The protocol for this trial and supporting CONSORT checklist are available as supporting information; see Checklist S1 and Protocol S1. This was a single centre, double blind, exploratory randomised controlled trial comparing oral ergocalciferol to placebo in patients with CKD stage 3–4 (estimated glomerular filtration rate (eGFR) 60–15 ml/min/1.73 m^2^) and concomitant vitamin D deficiency (defined as a serum 25 hydroxy vitamin D (25 (OH) D) level of <16 ng/ml). The study was conducted at the Royal London Hospital, UK, between 1/5/2009 and 1/9/2010. All patients provided written informed consent and ethical approval was obtained from the East London Research Ethics Committee. The trial was registered at clinicaltrials.gov (Clinical trials number- NCT00882401, http://clinicaltrials.gov/ct2/show/NCT00882401) and conducted in accordance with the Declaration of Helsinki.

### Inclusion/exclusion criteria

The principle inclusion criteria were an eGFR between 15 and 60 ml/min/1.73 m^2^ with evidence of 2 consecutive measures of eGFR <60 ml/min/1.73 m^2^ by the 4 variable MDRD equation [36] at least 3 months apart, serum 25 (OH) D levels <16 ng/ml, age >18 and <80 years, no evidence of diabetes mellitus (fasting blood sugar <128 mg/dl, not taking any diabetic medication), not receiving nor having had renal replacement therapy in the preceding three months

**The principle exclusion criteria were current vitamin D therapy of any type, serum calcium above 10.4 mg/dl (based on the upper limit of the laboratory reference range** [37] **), pregnant or lactating women, presence of conditions which predispose to hypercalcaemia, renal calculi and presence of a disease other than CKD associated with microcirculatory dysfunction.**

### Intervention and randomization

Patients were randomised to either ergocalciferol (Sanofi Aventis, New Jersey, USA) or a matching placebo. The dose of ergocalciferol was 50,000 IU weekly for one month followed by 50,000 IU monthly for 5 months resulting in a total dose of 450,000 IU over 6 months. This was in line with existing K/DOQI guidelines for the replacement of vitamin D in patients with CKD at the time the study was designed [37]. The dose of ergocalciferol was standardized for all patients to ensure equal dosing of ergocalciferol over the duration of the study to avoid the potentially confounding effect of varying doses of ergocalciferol based on initial serum concentrations of 25 (OH) D. The control arm received a matching placebo given at the same dose schedule as ergocalciferol. All patients received dietary advice appropriate to their stage of CKD from specialist renal dietitians which included advice on dietary intake of calcium, phosphate, sodium, potassium and protein. Patients were reviewed monthly for 6 months. A 2 week washout period for any vitamin D containing drugs or over the counter supplements was included before randomization, however no subjects required vitamin D washout. The randomization schedule was developed by an independent accountant. Sequentially numbered, sealed envelopes were used to achieve allocation concealment. Envelopes were stored and sequentially dispensed to study patients by the hospital clinical trial pharmacy who were blinded to the intervention and allocation as were the remainder of the study team.

### Study endpoints

The primary outcome measure was microcirculatory endothelial function assessed by laser Doppler flowmetry (LDF) over forearm skin after iontophoresis of acetylcholine (ACh). Secondary outcome measures were microvascular parameters including skin autofluorescence (AF) and side stream dark field imaging (SDF) of the sub lingual microcirculation as well as macrovascular parameters including blood pressure, pulse pressure, aortic pulse wave velocity (aPWV), left ventricular mass index (LVMI) and bone mineral parameters (see File S1 for details of all techniques).

### Clinical assessments

All patients were reviewed at the Royal London Hospital kidney unit. Patients were instructed to wear loose clothing, avoid caffeine and nicotine for 12 hours prior to assessments and rested for 15 minutes in a temperature and humidity controlled room before microcirculatory assessments.

### Iontophoresis and laser Doppler flowmetry

Iontophoresis involves the delivery of charged particles to the local microcirculation, through the skin, using electrically repulsive forces. Laser Doppler flowmetry is a non-invasive technique which uses the Doppler principle to measure flux of erythrocytes in sub dermal capillaries. Increasing red cell flux after iontophoresis reflects microcirculatory vasodilatation as a consequence of improved endothelial function. The combination of these techniques is a validated method for studying endothelial function in the microcirculation in various pathologies including CKD. [38], [39], [40] A 1% solution of both ACh and sodium nitroprusside (SNP) were iontophoresed on the volar aspect of the non-dominant forearm using a low current protocol to reduce galvanic effect [41] with a maximum achieved dose of 75 µA after 7.5 minutes. To eliminate baseline variability, relative percentage change from baseline flux after the maximum iontophoretic dose is the primary outcome measure (see Figure S1 and Table S1 in File S1) [38].

### Skin autofluorescence

Tissue advanced glycation end (AGE) product levels are an independent risk predictor of microcirculatory complications and are predictors of CVD in renal failure [42]. The technique utilises the AGE reader (Diagnoptics, Netherlands) which provides a non-invasive measure of AGE in the skin. AGE products correlate with measures of skin AF provided by the output from the AGE reader device [42].

### Side stream dark field imaging of the sublingual microcirculation

This is a non-invasive, real time, imaging tool to assess intra-vital capillary blood flow. An analysis of the moving cells in the recorded video images permits the quantitative measurement of red blood cell flow in the capillaries. SDF images were scored and interpreted according to standard guidelines [43].

### Macrocirculatory parameters

Aortic pulse wave velocity (Vicorder device, Skidmore Medical, UK) was measured to determine if changes in microcirculatory function occurred independently of changes in large conduit vessels. LVMI was measured by cardiac magnetic resonance imaging (cMRI) to determine if the hypothesized improved microcirculatory function resulted in reduced peripheral resistance leading to reduced cardiac workload and subsequent reductions in LVMI.

### Biochemical analysis

All biochemical tests were performed in our hospital laboratory by a Roche modular unit analyser (F. Hoffmann-La Roche Ltd, Switzerland). Serum vitamin D levels were assessed by UPLC-MS/MS, which is a quantitative ultra-performance liquid chromatography tandem mass spectrometry assay. Parathyroid hormone (PTH) levels were assessed by the Roche E170 intact PTH assay (Roche Diagnostics, Mannheim)

### Experimental work

Human aortic endothelial cells (HAEC, Promocell, UK) were cultured and incubated for 24 h using either low (12 ng/dl) or high (120 ng/dl) concentrations of ergocalciferol. Real time polymerase chain reaction (RT-PCR) was used to evaluate the effect of ergocalciferol on the expression of endothelial nitric oxide synthase (eNOS). Nitrite levels in cell supernatant, to evaluate the downstream effect of changes in eNOS expression in cells, were measured by a chemiluminescent technique [44] (see File S1 and figures S1-5 for full description of all techniques).

### Statistical analysis

At the time of designing the study, there was insufficient available evidence of the effect of ergocalciferol therapy on microcirculatory parameters to undertake a standard power calculation. Our hypothesis was that ergocalciferol would significantly improve the function of the endothelium in patients with CKD and concomitant vitamin D deficiency. Given the profoundly low levels of 25 (OH) D in this patient group, the expected rise in serum 25 (OH) D levels with ergocalciferol and predicted lack of change of 25 (OH) D levels in the placebo group, we estimated that 30% of patients in the placebo group and 80% of the patients in the ergocalciferol group would have an improvement in peripheral LDF measured by relative change from baseline flux after 6 months of therapy. At 80% power and with a significance level of 0.05, this required 19 patients per arm. An intention to treat analysis was performed.

### Clinical trial subjects

The two groups were compared for similarity at baseline and after 6 months of therapy using the Student's t test for normally distributed variables, Mann Whitney tests for non-parametric data and Chi squared tests or Fisher's exact test for proportions. Differences in 25 (OH) D levels and change from baseline flux measured by LDF after iontophoresis were analysed using a two way repeated measures ANOVA test followed by Bonferroni post tests for comparisons at pre determined time points (1, 3 and 6 months). We confirmed our findings using a mixed effects model. Forearm laser Doppler flowmetry is expressed as the percentage increase in flux from baseline after iontophoresis of ACh [38]. Four patients in the ergocalciferol group and 1 in the placebo group completed an initial 3 months on therapy before the end of the predetermined study period and this data was included in the analysis. The analysis of data at 6 months includes data from all remaining patients who completed the full follow up period. SDF imaging of the sublingual microcirculation was expressed as functional capillary density (mm^−1^) and microvascular flow index as described previously [43]. LVMI, SDF imaging, skin AF and bone mineral parameters were analysed using t tests and Mann Whitney tests based on the distribution of the data. A p value of <0.05 was considered statistically significant. Analysis was conducted on Stata version 10 (www.stata.com) and GraphPad Prism software version 5 (see File S1 and figures S2–S5 for sub-group analysis).

### Cell experiments

RT-PCR data were analyzed with ABI 7900HT Prism sequence detector software (SDS Version 2.3, Applied Biosystems) to determine differential gene expression for eNOS compared to β actin. Differences in nitrite levels were assessed using the Student t-test. Ethanol is included since this was used to dilute the ergocalciferol. Statistical analysis was performed using GraphPad Prism software (version 5).

## Results

**Figure 1:**
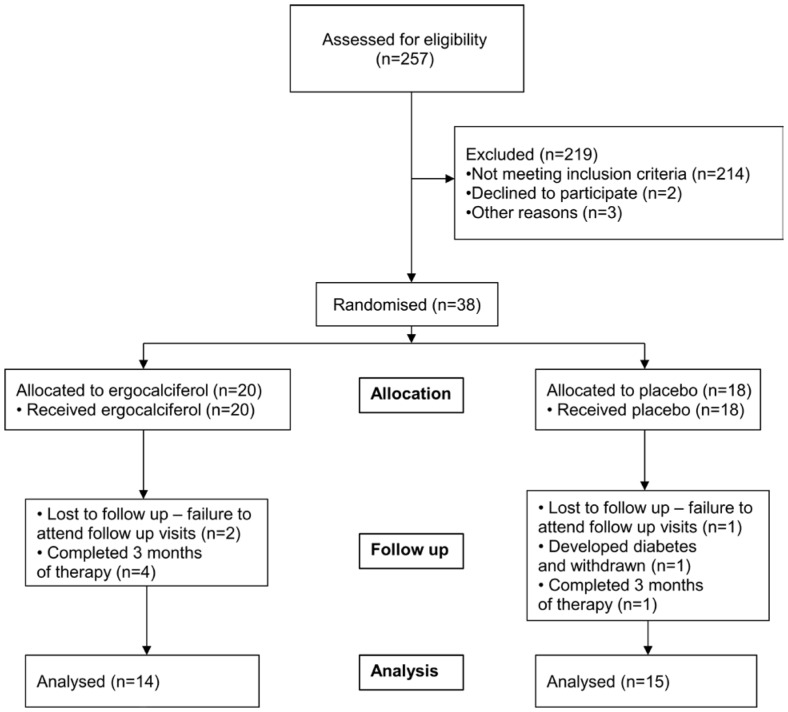
Recruitment schedule for study patients.

**Figure 2:**
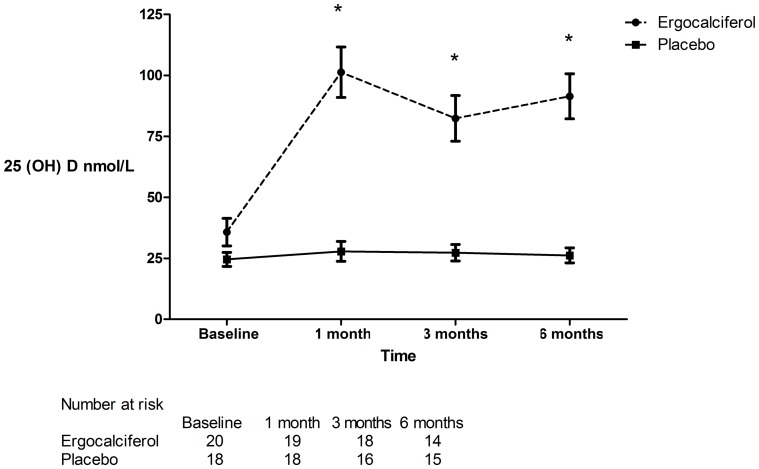
25 (OH) D levels in patients treated with ergocalciferol and placebo. Bonferroni post tests following two way repeated measures ANOVA at 1,3 and 6 months p<0.0001. (*  =  statistically significant).

Patient screening, enrolment and randomization are shown in figure 1. The treatment and placebo groups were similar with respect to demographic, clinical (table 1) and laboratory parameters (table 2). Two patients were lost to follow up in the ergocalciferol arm and 1 in the placebo arm. All patients self reported complete compliance and this was confirmed by manual inspection of study medication bottles at each visit. No patients were taking nitrate containing medications that may have acted as vasodilators through the donation of nitric oxide. After 6 months of treatment with ergocalciferol, 25 hydroxy vitamin D (25 (OH) D) levels increased significantly in the treatment group (p<0.0001) (figure 2) but there were no significant changes in other biochemical parameters (table 3).

**Table 1:** Baseline demographic and clinical data for patients.

	Ergocalciferol (n = 20)	Placebo (n = 18)	p value
**Age years**	45.8 (10.0)	48.8 (12.2)	0.39
**Sex (% male)**	14 (60.9%)	14 (73.7%)	0.22
**Body mass index**	30.4 (7.1)	29.2 (3.4)	0.51
**Ethnicity**			0.57
**Caucasian**	5 (21.8%)	6 (31.6%)	
**Non-Caucasian**	15 (78.2%)	12 (68.4%)	
**Cause of CKD**			0.74
**Hypertension**	5 (25%)	7 (38.9%)	
**Glomerulonephritis**	8 (40%)	5 (27.8%)	
**ADPKD**	2 (10%)	1 (5.6%)	
**Other** ∼	5 (25%)	5 (27.7%)	
**Smoking status**			0.59
**Current smoker**	1 (5%)	2 (11.1%)	
**Never/Ex-smoker**	19 (95%)	16 (88.9%)	
**Presence of endovascular stent devices**	0 (0%)	1 (5.6%)	0.47
**ACE-I/ARB**	16 (80%)	12 (66.7%)	0.33
**β Blocker**	7 (35%)	6 (33.3%)	0.57
**Statin use**	9 (45%)	7 (38.9%)	0.84
**Anti platelet therapy**	2 (10%)	3 (16.7%)	0.48
**Folic acid**	1 (5.0%)	1 (5.6%)	1.00
**Nitrate containing medications** #	0 (0%)	0 (0%)	1.00
**Medications containing Vitamin D**	0 (0%)	0 (0%)	1.00
**BP in past history**	15 (65.2%)	11 (57.9%)	0.63
**Systolic BP (mmHg)**	114 (10)	119 (10)	0.11
**Diastolic BP (mmHg)**	70 (8)	71 (7)	0.57
**MAP (mmHg)**	84 (8)	87 (8)	0.29
**Pulse pressure (mmHg)**	45 (7)	48 (6)	0.08
**aPWV (m/s)**	8.5 (1.1)	8.5 (1.5)	0.66
**LVMI (g/m2)**	96.1 (36.3)	87.5 (174)	0.55
**Skin AF (AU)**	2.8 (0.9)	3.1 (0.9)	0.26
**FCD (mm-1)**	5.2 (0.5)	5.0 (0.5)	0.78
**MFI (AU)**	2.5 (0.1)	2.4 (0.1)	0.54

Figures in brackets are standard deviation of the mean or % of total in treatment group. ADPKD – autosomal dominant polycystic kidney disease. BP  =  blood pressure, ACE-I angiotensin converting enzyme inhibitor, ARB – angiotensin receptor blocker, MAP  =  mean arterial pressure, aPWV  =  aortic pulse wave velocity, LVMI – left ventricular mass index, AF – auto fluorescence, FCD – functional capillary density, MFI – microvascular flow index. AU – arbitrary units

# - any form of glyceryl trinitrate, isosorbide mononitrate, isosorbide dinitrate or other esters of nitric acid

∼ - Additional causes of CKD in ergocalciferol group: tubulo-interstitial nephritis (n = 1), reflux nephropathy (n = 2), unknown (n = 2). Placebo group: reflux nephropathy (n = 2), ischaemic nephropathy presumed due to reno-vascular disease (n = 1), unknown (n = 2).

**Table 2:** Baseline laboratory data for CKD patients randomised to either ergocalciferol or placebo.

	Ergocalciferol (n = 20)	Placebo (n = 18)	p value
**Creatinine (mg/dl)**	2.3 (0.8)	2.0 (1.0)	0.60
**eGFR (ml/min/1.73 m^2^)**	33.0 (13.5)	38.7 (15)	0.39
**Stage of CKD**			0.33
**Stage 3**	9 (45%)	13 (72.2%)	
**Stage 4**	11 (55%)	5 (27.8%)	
**Hb (g/dl)**	12.8 (1.8)	12.6 (1.4)	0.63
**Calcium (mg/dl)**	8.8 (0.8)	8.8 (0.8)	0.74
**Phosphate (mg/dl)**	3.8 (0.6)	3.5 (0.6)	0.16
**Calcium phosphate product (mg^2^/dl^2^)**	33.0 (4.2)	31.2 (5.1)	0.25
**PTH (pg/L)**	102.8 (76.4)	118.9 (103.8)	0.60
**CRP (mg/L)**	7.6 (17.2)	5.9 (9.8)	0.71
**Urine P:CR**	190.8 (276.4)	102.7 (147.0)	0.32
**Total cholesterol (mg/dl)**	201 (53)	185 (39)	0.36
**High density lipoprotein cholesterol (mg/dl)**	57.5 (24.0)	46.7 (15.9)	0.20

Figures in brackets are standard deviation of the mean. eGFR  =  estimated glomerular filtration rate, 25 (OH) D  = 25 hydroxy vitamin D, Hb  =  haemaglobin, PTH  =  parathyroid hormone, CRP  =  C reactive protein, P:CR  =  protein:creatinine ratio.

**Table 3:** Laboratory results in both groups after 6 months of therapy.

	Ergocalciferol (n = 14)	Placebo (n = 15)	p value
**Creatinine (mg/dl)**	2.4 (0.9)	2.3 (1.1)	0.80
**eGFR (ml/min/1.73 m^2^)**	31.4 (10.6)	35.0 (14.5)	0.44
**Hb (g/dl)**	12.6 (2.1)	12.4 (1.3)	0.73
**Calcium (mg/dl)**	9.1 (0.7)	8.9 (0.6)	0.43
**Phosphate (mg/dl)**	3.7 (0.74)	3.7 (1.2)	0.98
**Calcium phosphate product (mg^2^/dl^2^)**	33.6 (8.2)	32.6 (3.5)	0.66
**PTH (pg/L)**	97.2 (74.5)	135.8 (96.2)	0.26
**CRP (mg/L)**	7.5 (15.0)	9.7 (19.8)	0.76
**Urine P:CR**	154.0 (210.3)	117.5 (126.3)	0.62
**Total cholesterol (mg/dl)**	193 (38)	174 (35)	0.21
**High density lipoprotein cholesterol (mg/dl)**	54.2 (25.2)	50.8 (15.3)	0.67

Figures in brackets are standard deviation of the mean. eGFR  =  estimated glomerular filtration rate, 25 (OH) D  = 25 hydroxy vitamin D, Hb  =  haemaglobin, PTH  =  parathyroid hormone, CRP  =  C reactive protein, P:CR  =  protein:creatinine ratio.

### The effect of ergocalciferol on microcirculatory parameters

**Figure 3:**
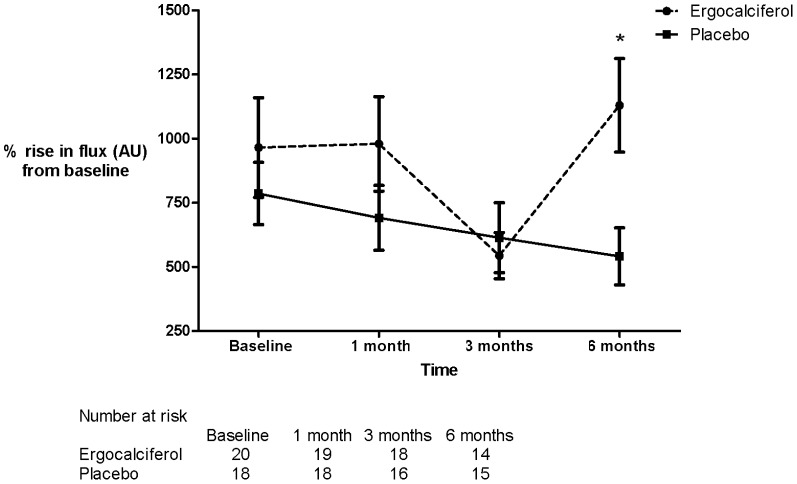
Percentage rise from baseline flux in arbitrary units (AU) after iontophoresis of ACh. Absolute values of percentage change in flux (AU): baseline - ergocalciferol 964.8, placebo 785.9 (p = NS). 1 month - ergocalciferol 979.5, placebo 690.9 (p = NS). 3 months – ergocalciferol 543.7, placebo 613.5 (p = NS). 6 months – ergocalciferol 1130.0, placebo 540.6 (p = 0.012). p values are Bonferroni post test following two way repeated measures ANOVA. (*  =  statistically significant).

**Figure 4:**
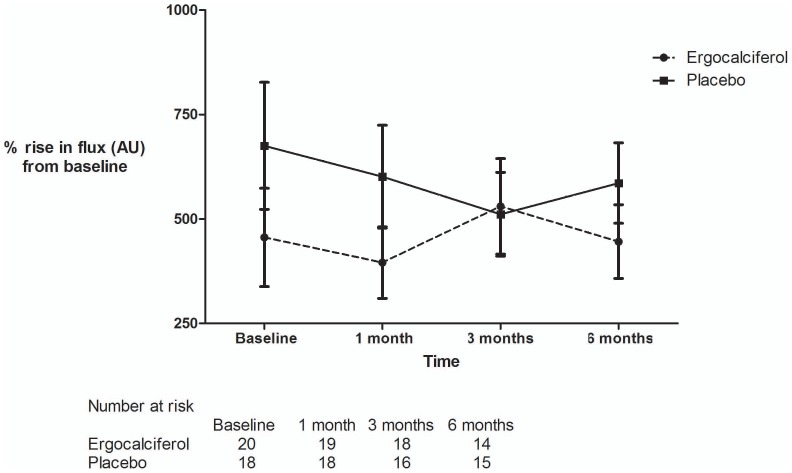
Percentage rise from baseline flux in arbitrary units (AU) after iontophoresis of SNP. Absolute values of percentage change in flux (AU): baseline - ergocalciferol 455.8, placebo 670.1 (p = NS). 1 month - ergocalciferol 395.5, placebo 601.3 (p = NS). 3 months – ergocalciferol 530.2, placebo 511.2 (p = NS). 6 months – ergocalciferol 445.7, placebo 585.9 (p = NS). p values are Bonferroni post test following two way repeated measures ANOVA.

Treatment with ergocalciferol compared to placebo was associated with a significant increase in change from baseline flux measured by LDF after iontophoresis of ACh (repeated measures 2 way ANOVA p = 0.03) with a significant difference between treatment groups observed at 6 months (Bonferroni post test p = 0.012) (figure 3). There were no significant differences in change from baseline flux after the iontophoresis of SNP between treatment groups at 6 months (repeated measures 2 way ANOVA p = 0.18) (figure 4). The use of a mixed effects model did not change the significance of these findings (change from baseline after iontophoresis at 6 months follow up: ACh p = 0.03, SNP p = 0.36).

Skin AF was significantly lower in patients treated with ergocalciferol after 6 months, reflecting reduced levels of tissue oxidative stress (p = 0.03). At the end of the follow up period, there were no differences in parameters of functional capillary density (FCD) or microvascular flow index (MFI) assessed by SDF imaging of the sublingual microcirculation (table 4).

**Table 4:** Measures of macrovascular parameters in both groups after 6 months of therapy.

	Ergocalciferol (n = 14)	Placebo (n = 15)	p value
**Systolic BP (mmHg)**	118 (10)	123 (15)	0.26
**Diastolic BP (mmHg)**	74 (6)	70 (9)	0.15
**MAP (mmHg)**	89 (7)	88 (10)	0.77
**Pulse pressure (mmHg)**	44 (8)	53 (12)	0.01
**aPWV (m/s)**	8.4 (1.3)	8.5 (1.2)	0.78
**LVMI (g/m^2^)**	94.7 (28.4)	110 (54.3)	0.44
**Skin AF (AU)**	2.8 (0.6)	3.5 (0.9)	0.03
**FCD (mm^−1^)**	5.3 (0.7)	5.4 (0.8)	0.90
**MFI (AU)**	2.4 (0.2)	2.4 (0.2)	0.81

Figures in brackets are standard deviation of the mean. BP  =  blood pressure, MAP  =  mean arterial pressure, aPWV  =  aortic pulse wave velocity, LVMI – left ventricular mass index, AF – auto fluorescence, FCD – functional capillary density, MFI – microvascular flow index. AU – arbitrary units.

### The effect of ergocalciferol on macrocirculatory parameters

Pulse pressure was significantly lower in patients treated with ergocalciferol after 6 months (p = 0.01) but systolic, diastolic and mean arterial pressures were similar between treatment groups. There were no differences between the study groups in aPWV or LVMI after 6 months of therapy (table 4).

### Safety data

There were no serious adverse events reported. Three patients in each group experienced episodes of gout that resolved with analgesia. The highest 25 (OH) D level recorded was 68.5 ng/ml. There were no recorded episodes of hypercalcaemia (defined as a serum calcium >10.4 mg/dl).

### The effect of ergocalciferol on eNOS expression and nitrite levels in cultured human aortic endothelial cells

**Figure 5:**
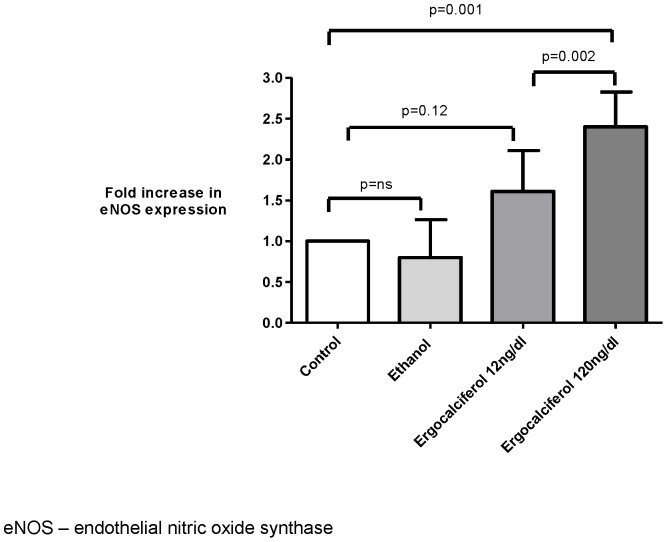
Fold increase in eNOS expression by RT-PCR in cultured HAEC.

**Figure 6:**
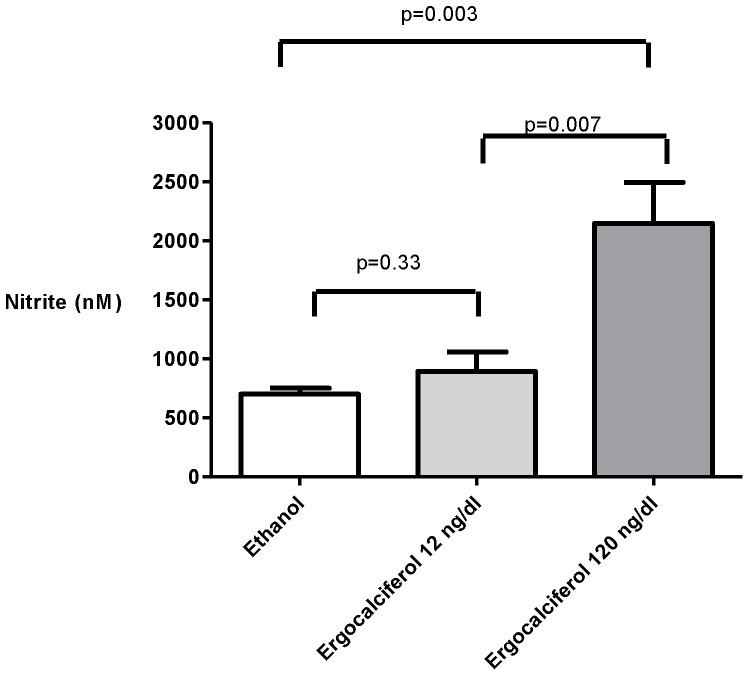
Nitrite levels in supernatants of HAEC. Cultured in low dose (12 ng/dl) and high dose (120 ng/dl) ergocalciferol after 24 h incubation.

RT-PCR analysis for the fold-increase of eNOS compared to β-actin as a control gene demonstrated a dose dependent increase in expression with a 2.4-fold increase in eNOS expression after 24 h of incubation with high concentration ergocalciferol (120 ng/dl) compared to a 1.6 fold increase with low dose ergocalciferol (12 ng/dl) (p = 0.002). Only high concentration ergocalciferol resulted in a statistically significant increase in fold expression of eNOS compared to the control experiment (p<0.001) (figure 5). There was no significant difference in nitrite levels between control and low dose ergocalciferol treated cells at 24 h. However, high dose ergocalciferol treatment produced a significant rise in nitrite compared to both low dose (p = 0.007) and vehicle control (p = 0.003) (figure 6).

## Discussion

In this study, ergocalciferol therapy over 6 months in patients with CKD and concomitant vitamin D deficiency was associated with improved endothelium dependent microcirculatory function and a reduction in measures of tissue oxidative stress. The increase in relative change of flux from baseline after iontophoresis with ACh but not SNP in subjects treated with ergocalciferol indicates improved microcirculatory function occurred through an endothelium dependent mechanism. Reduction in measures of skin AF in the ergocalciferol group correlates with reduced oxidative stress in these patients which will contribute to improved endothelial function and a reduced risk of future CV events [42]. This is in line with previous experimental work that demonstrated the protective effect of calcitriol in human endothelial cells cultured with AGE products [21].

In our study, bone mineral parameters, kidney function, C reactive protein, blood pressure and aPWV were similar at 6 month follow up suggesting that functional changes to the microcirculation occurred independently of these parameters, adding strength to the argument that ergocalciferol may have a specific mechanism of action within the microcirculation. In contrast to other studies [45], [46], proteinuria was unaffected in the ergocalciferol group although the differences in vitamin D compounds, dose schedule, study duration and populations between those studies and ours may explain this difference. aPWV did not decrease in line with the reduction in pulse pressure and this finding may reflect the short duration of the study. Studies with a longer follow up duration are more likely to demonstrate a fall in pulse wave velocity which may occur after a reduction in pulse pressure

After 6 months of therapy with ergocalciferol, there was no increase in the functional capillary density or flow within the microcirculation. This implies that the observed improvements in endothelium dependent microcirculatory function did not involve the recruitment of extra functionally relevant capillaries or changes in blood flow but rather that the endothelium dependent function of the existing microcirculatory network was improved by the direct effect of ergocalciferol.

This hypothesis is supported by the results of parallel *in vitro* experiments in which ergocalciferol, in a dose dependent manner, upregulated eNOS expression measured by RT-PCR. Nitric oxide generation compared to vehicle was numerically but not statistically significantly increased with low dose ergocalciferol but was significantly increased with high dose ergocalciferol. This suggests that higher doses of ergocalciferol are required to increase the functional effect of ergocalciferol on the endothelium and therefore that there may be a threshold 25 (OH) D concentration above which the maximum effects of ergocalciferol on the microcirculation are achieved.

The present study is the first of its kind to explore the effect of vitamin D on microcirculatory function in patients with CKD and concomitant vitamin D deficiency. The exclusion of patients with diabetes mellitus allowed us to evaluate the effect of ergocalciferol on the microcirculation in CKD without the potentially confounding effect of diabetes on endothelial function.

Prospective studies of the effect of vitamin D therapy in patients with CKD have so far failed to show a beneficial effect of vitamin D on endpoints including LVMI, aPWV, blood pressure and inflammatory biomarkers. [27], [47] The effect of nutritional vitamin D compounds on endothelial biomarkers and conduit artery endothelial function in kidney disease has been evaluated in two studies which have produced conflicting results. Marckmann *et al*. [27] compared the effect of 8 weeks of 40,000 IU of weekly cholecalciferol compared to a placebo in patients with both CKD and ESKD treated with haemodialysis. Patients in the control and intervention group were similar at baseline including cause of kidney disease, dialysis status and serum 25 (OH) D, 1,25 (OH)_2_ D_3_ and bone mineral parameters (PTH, Ca, PO_4_) levels. There was a significant increase in 25 (OH) D levels in the treatment compared to control group (154.7 nmol/L vs 23.5 nmol/L) and a significant increase in 1,25 (OH)_2_ D_3_ levels although the increase in 1,25 (OH)_2_ D_3_ levels was higher in non-dialysis compared to dialysis requiring CKD (median increase in the CKD group 49 pmol/L (p<0.01) compared to 14 pmol/L in the dialysis group (p>0.05)). After 8 weeks of treatment, PTH fell significantly in non-dialysis CKD patients but not in dialysis requiring CKD patients. Despite the significant increase in vitamin D levels in the cholecalciferol group, there was no reduction in markers of endothelial dysfunction including D-dimer, von Willebrand factor, fibrinogen and interleukin (IL) 8 or C reactive protein. Additionally, blood pressure, aPWV and aortic augmentation index did not change between the two groups. Specifically, there were no significant differential effects on these parameters when comparing cholecalciferol treated and untreated patients in the dialysis and non-dialysis groups.

Assimon *et al*. [26] have evaluated the effect of ergocalciferol (n = 20 on ergocalciferol for a mean of 39.2 weeks and n = 20 not receiving ergocalciferol) on markers of vascular endothelial adhesion in a case control study of 40 patients undergoing maintenance haemodialysis. There were no significant differences in baseline parameters including dialysis vintage and both groups were receiving an equivalent dose of doxercalciferol. Serum 25 (OH) D levels were higher in the ergocalciferol group (90.8 nmol/L compared to 60.2 nmol/L, p = 0.03). In the ergocalciferol group, there was reduction in levels of vascular adhesion molecules sVCAM-1, sICAM-1, P-selectin and in all patients there was a significant negative correlation between serum 25 (OH) D levels and P-selectin and E-selectin. There was no difference in inflammatory biomarkers including IL-6 and TNF-α. However, the functional response of the endothelium was not evaluated and the case control design of this study means that the causal relationship between endothelial adhesion molecules and ergocalciferol cannot be established.

Despite the prompt and sustained rise in 25 (OH) D levels in our patients, significant differences in key microcirculatory end points were only observed after 6 months of therapy with ergocalciferol even though 25 (OH) D levels were similar at 1,3 and 6 months. Previous clinical studies using high dose ergocalciferol or cholecalciferol in healthy and diabetic patients without significant kidney disease demonstrated improved microcirculatory function between 8–12 weeks [24], [25], [48]. The delay in attainment of significantly improved microvascular function in the current study and lack of improvement over 8 weeks in Marckmann *et al*. study [27] may be a consequence of the uraemic milieu reducing the response of the microcirculation both to the upregulation of eNOS [49] and its downstream effects [50] in increasing availability of vasodilator moieties.

The findings from our study suggest that treatment with high dose ergocalciferol over an extended period of time is required before there is an improvement in microcirculatory endothelial function. This concept will have important implications for determining both the optimum duration of therapy of ergocalciferol and the optimum serum 25 (OH) D level to ensure a maximally beneficial effect on the microcirculation.

The strengths of our study are its double blind randomised placebo control design, replacement of vitamin D in line with international guidelines [37] that was standardised for all patients and commensurate with baseline serum concentrations of 25 (OH) D as well as the use of techniques that specifically assessed both conduit artery and microcirculatory endothelial function. At the time of designing this study, microcirculatory endothelial function had not previously been evaluated in patients with CKD and concomitant VDD in a clinical trial setting. Iontophoresis has been used in the setting of clinical trials to evaluate endothelial function. [51], [52], [53], [54] The experimental conditions and iontophoretic protocol in the present study were standardised and changes in endothelial function were compared with baseline prior to treatment with ergocalciferol. The use of a low current iontophoresis protocol will have reduced the direct galvanic effect from the iontophoretic process on the endothelium seen when a higher current is used. [41] Therefore, any change seen in LDF after iontophoresis must be due to the direct effect of ergocalciferol itself on microvascular endothelial function.

Limitations of this study include the short follow up time and small sample size. The study duration is insufficient to detect significant differences between treatment groups in key outcome measures including CV events. Excluding patients with diabetes mellitus has limited the external validity but improved the internal validity and precision of the present study. Human aortic endothelial cells were not cultured in media consistent with the degree of CKD in the clinical trial subjects due to the complexity of establishing a culture medium that accurately reflects the earlier rather than more advanced stages of CKD. Consequently, the results from the *in vitro* experiments cannot be directly generalised to the uraemic milieu associated with CKD stage 3–4. The current study did not assess the effect of ergocalciferol on endothelial progenitor cells which are important mediators of endothelial repair and function and are reduced in patients at high risk of CVD [55]. Additional studies are required to address the effect of ergocalciferol on endothelial cells cultured in a medium more representative of the earlier stages of CKD as well as the effect of ergocalciferol on EPC number and function in CKD stage 3–4.

## Conclusions

High dose ergocalciferol therapy over 6 months improved microcirculatory function and reduced tissue oxidative stress in patients with CKD stage 3–4 and concomitant vitamin D deficiency. *In vitro* studies suggest this effect is mediated through increased expression of eNOS and greater generation of nitric oxide. The primary endpoint of the study reflects global vascular health [14] and it is therefore logical to consider that the observed improvements in microcirculatory function will translate into improved clinical outcomes including a reduction in CV events.

To test this hypothesis, studies in patients with CKD and concomitant vitamin D deficiency with longer follow up and adequately powered to detect CV end points are now required to determine both if improved microcirculatory endothelial function through ergocalciferol therapy subsequently reduces CV endpoints and to determine the optimum serum level of 25 (OH) D to maximize microvascular endothelial function.

## Supporting Information

Figure S1 Examples of iontophoresis dose response curves after 6 months of treatment for 2 patients after delivery of ACh demonstrating greater relative increase from baseline in flux in the ergocalciferol compared to placebo treated patient. Flux measured in arbitrary units (AU). Epoch reflects sequential dose increments of the iontophoretic protocol.
(TIF)

Figure S2 Percentage rise in flux from baseline after iontophoresis of ACh in patients with hypertension. Absolute values of percentage change in flux (AU): baseline - ergocalciferol 505.9, placebo 889.2 (p = NS). 1 month - ergocalciferol 853.5, placebo 1051.0 (p = NS). 3 months – ergocalciferol 519.2, placebo 1103.0 (p = NS). 6 months – ergocalciferol 671.6, placebo 1024.0 (p = NS). p values are Bonferroni post test following two way repeated measures ANOVA.
(TIF)

Figure S3 Percentage rise in flux from baseline after iontophoresis of SNP in patients with hypertension. Absolute values of percentage change in flux (AU): baseline - ergocalciferol 339.7, placebo 675.1 (p = NS). 1 month - ergocalciferol 866.9, placebo 724.0 (p = NS). 3 months – ergocalciferol 573.2, placebo 735.0 (p = NS). 6 months – ergocalciferol 682.2, placebo 579.0 (p = NS). p values are Bonferroni post test following two way repeated measures ANOVA.
(TIF)

Figure S4 Percentage rise in flux from baseline after iontophoresis of ACh in patients with glomerulonephritis. Absolute values of percentage change in flux (AU): baseline - ergocalciferol 762.9, placebo 1220.0 (p = NS). 1 month - ergocalciferol 1141.0, placebo 483.8 (p = NS). 3 months – ergocalciferol 647.7, placebo 1023.0 (p = NS). 6 months – ergocalciferol 1086, placebo 661.8 (p = NS). p values are Bonferroni post test following two way repeated measures ANOVA.
(TIF)

Figure S5 Percentage rise in flux from baseline after iontophoresis of SNP in patients with glomerulonephritis. Absolute values of percentage change in flux (AU): baseline - ergocalciferol 794.2, placebo 637.4 (p = NS). 1 month - ergocalciferol 690.6, placebo 708.8 (p = NS). 3 months – ergocalciferol 455.6, placebo 830.8 (p = NS). 6 months – ergocalciferol 387.3, placebo 504.1 (p = NS). p values are Bonferroni post test following two way repeated measures ANOVA.
(TIF)

Protocol S1 Full study protocol.
(DOCX)

Checklist S1 Study CONSORT check list.
(DOCX)

File S1 Full methodology. Contains Table S1, full iontophoresis protocol.
(DOCX)
