# Supplementary figures and images for: Ergocalciferol and Microcirculatory Function in Chronic Kidney Disease and Concomitant Vitamin D Deficiency: An Exploratory, Double Blind, Randomised Controlled Trial
Source: PLoS One. 2014 Jul 9;9(7):e99461. doi: 10.1371/journal.pone.0099461 (PMC4090117; doi:10.1371/journal.pone.0099461)

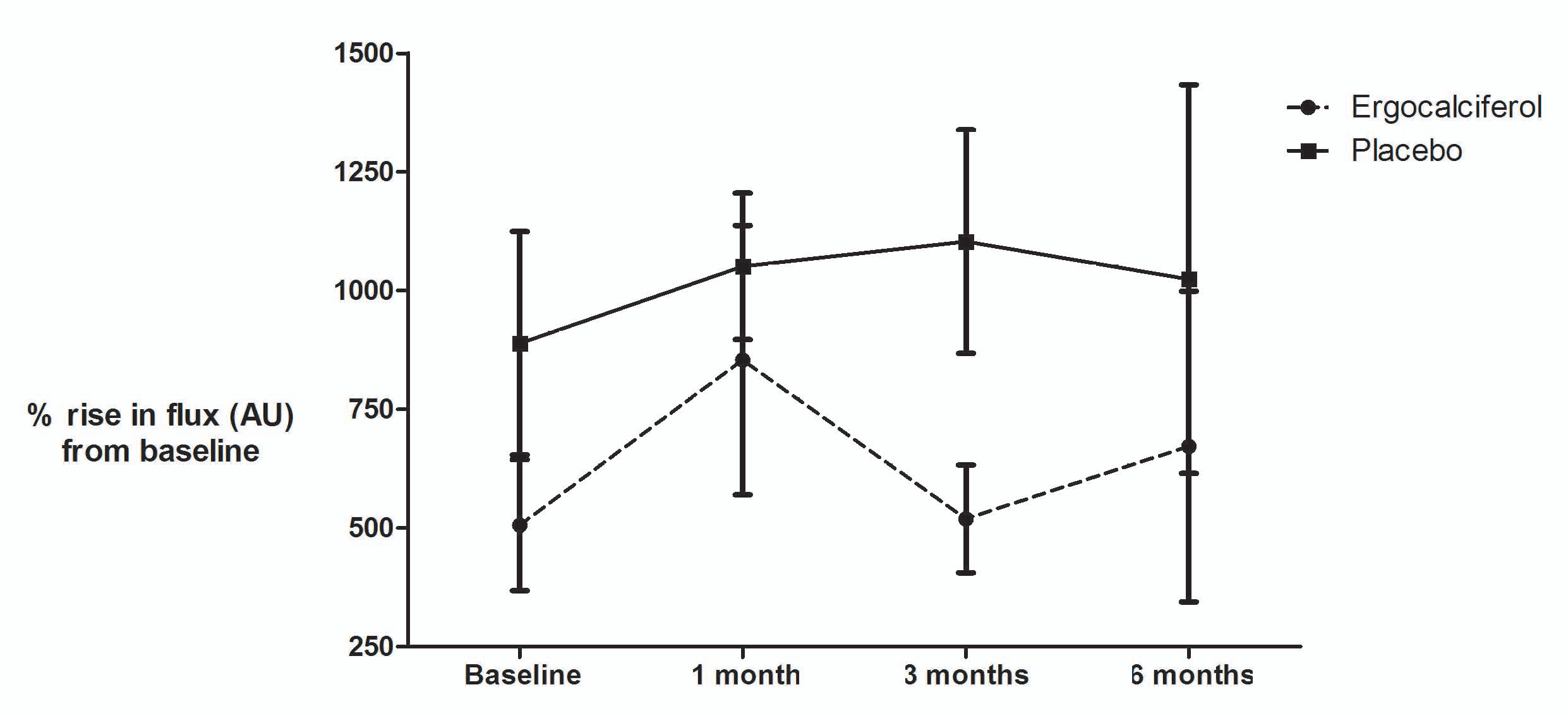

Supplement: Figure S2 — Percentage rise in flux from baseline after iontophoresis of ACh in patients with hypertension. Absolute values of percentage change in flux (AU): baseline - ergocalciferol 505.9, placebo 889.2 (p = NS). 1 month - ergocalciferol 853.5, placebo 1051.0 (p = NS). 3 months – ergocalciferol 519.2, placebo 1103.0 (p = NS). 6 months – ergocalciferol 671.6, placebo 1024.0 (p = NS). p values are Bonferroni post test following two way repeated measures ANOVA. (TIF) [file pone.0099461.s002.tif]

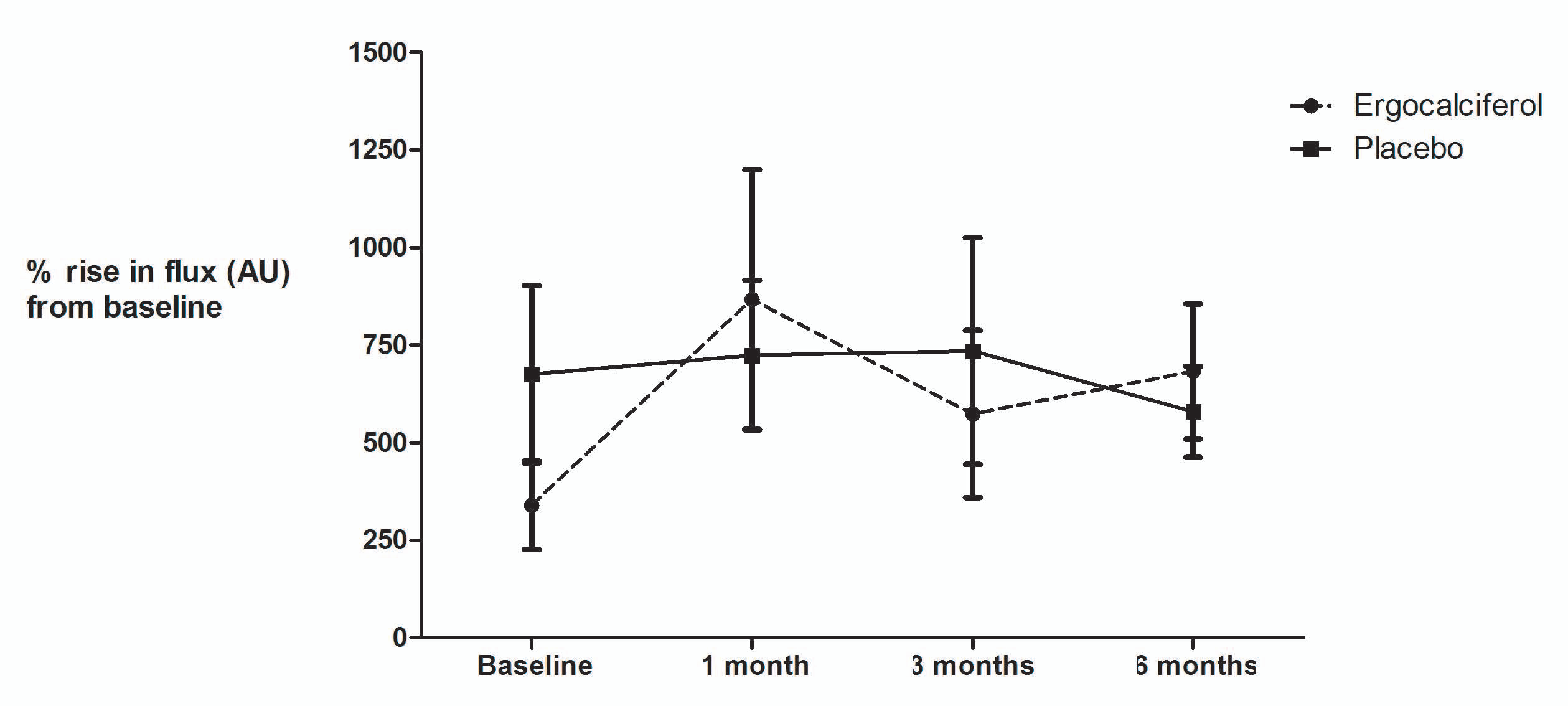

Supplement: Figure S3 — Percentage rise in flux from baseline after iontophoresis of SNP in patients with hypertension. Absolute values of percentage change in flux (AU): baseline - ergocalciferol 339.7, placebo 675.1 (p = NS). 1 month - ergocalciferol 866.9, placebo 724.0 (p = NS). 3 months – ergocalciferol 573.2, placebo 735.0 (p = NS). 6 months – ergocalciferol 682.2, placebo 579.0 (p = NS). p values are Bonferroni post test following two way repeated measures ANOVA. (TIF) [file pone.0099461.s003.tif]

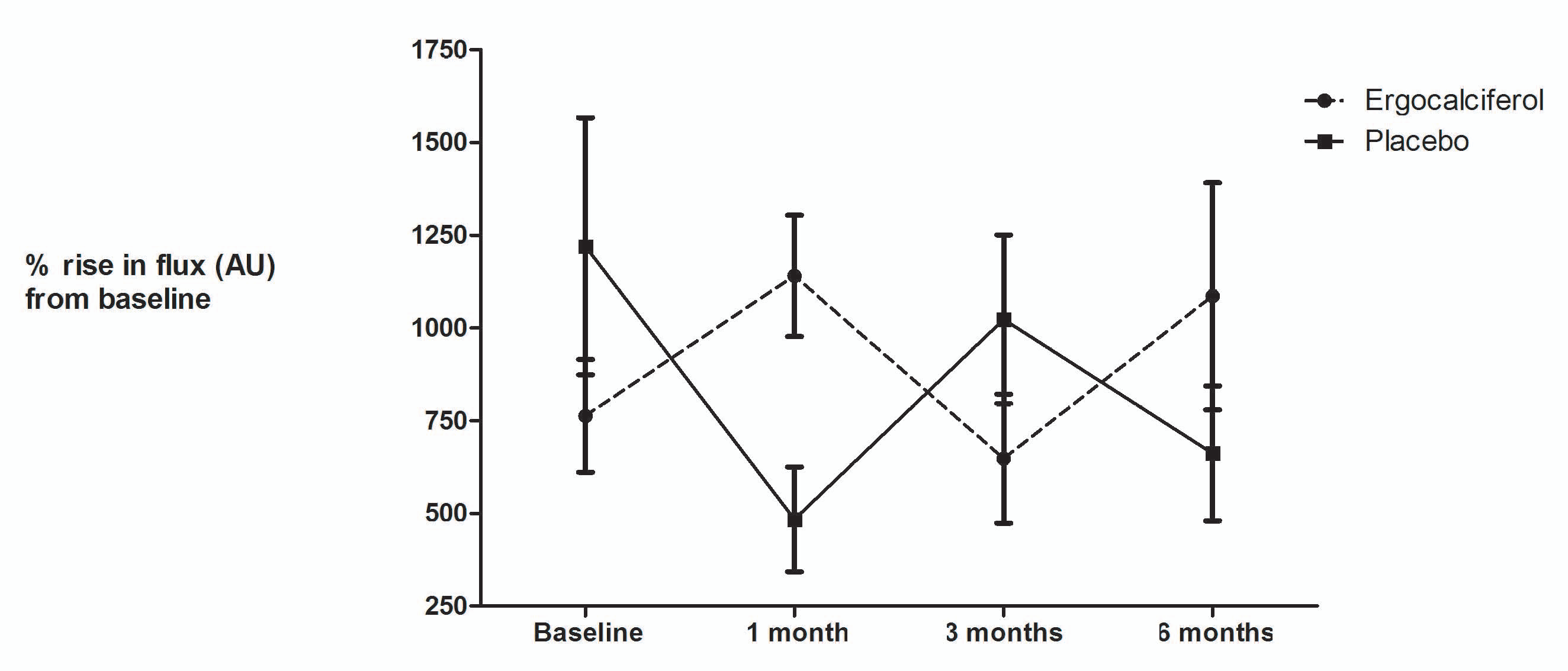

Supplement: Figure S4 — Percentage rise in flux from baseline after iontophoresis of ACh in patients with glomerulonephritis. Absolute values of percentage change in flux (AU): baseline - ergocalciferol 762.9, placebo 1220.0 (p = NS). 1 month - ergocalciferol 1141.0, placebo 483.8 (p = NS). 3 months – ergocalciferol 647.7, placebo 1023.0 (p = NS). 6 months – ergocalciferol 1086, placebo 661.8 (p = NS). p values are Bonferroni post test following two way repeated measures ANOVA. (TIF) [file pone.0099461.s004.tif]

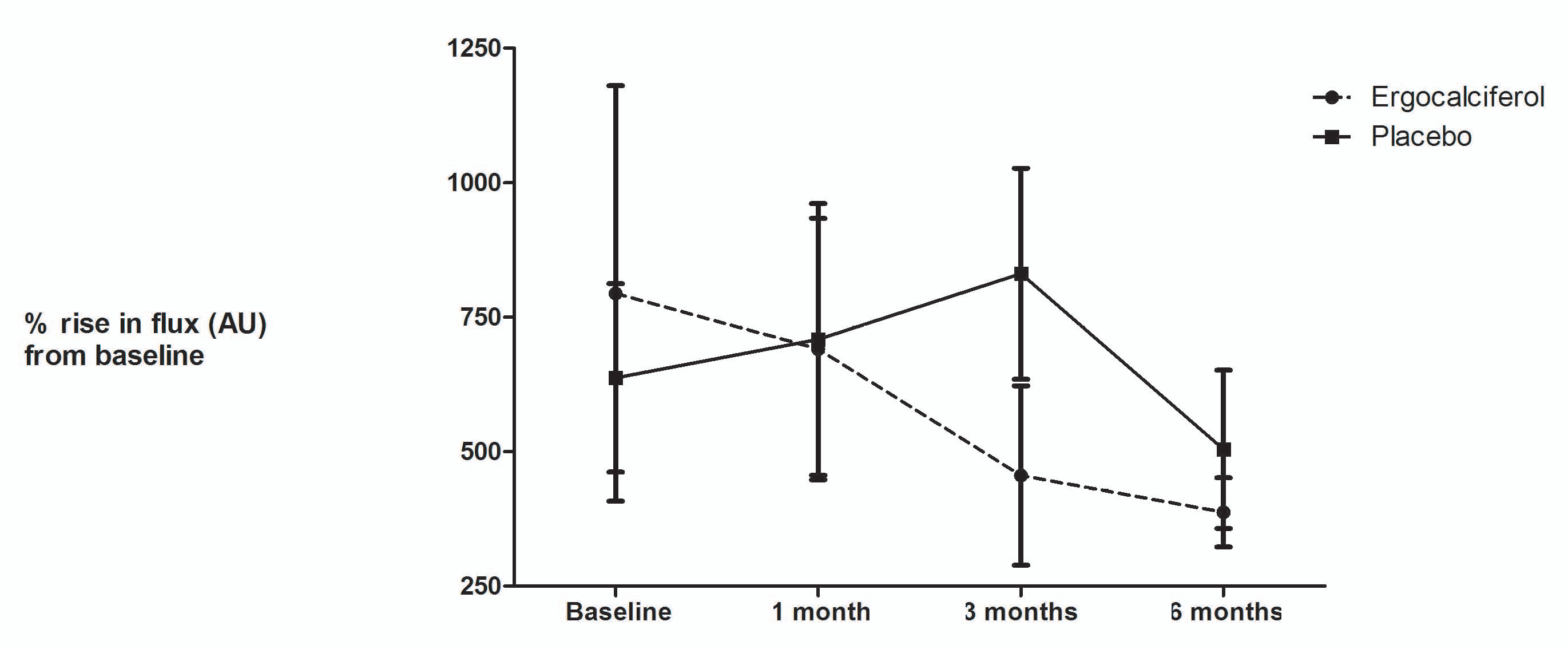

Supplement: Figure S5 — Percentage rise in flux from baseline after iontophoresis of SNP in patients with glomerulonephritis. Absolute values of percentage change in flux (AU): baseline - ergocalciferol 794.2, placebo 637.4 (p = NS). 1 month - ergocalciferol 690.6, placebo 708.8 (p = NS). 3 months – ergocalciferol 455.6, placebo 830.8 (p = NS). 6 months – ergocalciferol 387.3, placebo 504.1 (p = NS). p values are Bonferroni post test following two way repeated measures ANOVA. (TIF) [file pone.0099461.s005.tif]
