# Supplementary material for: Ergocalciferol and Microcirculatory Function in Chronic Kidney Disease and Concomitant Vitamin D Deficiency: An Exploratory, Double Blind, Randomised Controlled Trial
Source: PLoS One. 2014 Jul 9;9(7):e99461. doi: 10.1371/journal.pone.0099461 (PMC4090117; doi:10.1371/journal.pone.0099461)
Supplement: Protocol S1 — Full study protocol. (DOCX) [file pone.0099461.s006.docx]

**The effect of vitamin D on the microcirculation of patients with chronic kidney disease and vitamin d deficiency**

A single centre, double blind, randomised controlled trial comparing oral ergocalciferol and placebo

**Investigator**

Dr Gavin Dreyer

Dept of translational medicine and therapeutics

John Vane Science Building

William Harvey Research Institute (WHRI)

Charterhouse Square

London

EC1M 6BQ

Tel - 07779652081, Gavin.Dreyer@bartsandthelondon.nhs.uk

MB ChB (Hons) MRCP

**Funder**

Barts and the London Trust

Royal London Hospital

Whitechapel Road

London

E1 1BB

Contact: Dr Alistair Chesser, department head

**Research Team Members**

Professor M.M. Yaqoob and Dr Gavin Dreyer

Translational medicine and therapeutics

John Vane Science Building

William Harvey Research Institute

Charterhouse Square

London

EC1M 6BQ

0207 3777236, [m.m.yaqoob@qmul.ac.uk](mailto:m.m.yaqoob@qmul.ac.uk), Gavin.Dreyer@bartsandthelondon.nhs.uk

**Sponsor**

Barts and the London NHS Trust Gerry Leonard Head of Resources R&D Department

24-26 Walden Street, Whitechapel, London,E1 2ANPhone: 0207 882 7260

Email: gerry.leonard@bartsandthelondon.nhs.uk

**Trust(s) where research will take place**

Barts and the London NHS Trust

INVESTIGATOR SIGNATURE __________________ DATE _____________

**Submission reference: 15090/20517/1/897**

| **Date submitted to R&D Office:** 05/03/2009 | **EudraCT No:**  2008-008745-38 |
| --- | --- |
| **Date Submitted to REC:** 05/03/2009 | **Protocol Number.**  CKD-VitD-microcirc |
|  | |

**Principal Investigator**:

Dr Gavin Dreyer

Department of translational medicine

John Vane Science Building

William Harvey Research Institute

Charterhouse Square

London

EC1M 6BQ

Tel - 07779652081

Gavin.Dreyer@bartsandthelondon.nhs.uk

**Chief Investigator**

Professor M.M. Yaqoob and Dr Gavin Dreyer

Translational medicine and therapeutics

John Vane Science Building

William Harvey Research Institute

Charterhouse Square

London

EC1M 6BQ

0207 3777236, m.m.yaqoob@qmul.ac.uk

**Monitors:**

Dr Martin Raftery

Renal Unit

Royal London Hospital

12 Whitechapel Road

London

E1 1BB

Dr Chris Thiemermann

Centre for Translational Medicine and Therapeutics
Barts and The London, Queen Mary's School of Medicine and Dentistry
2nd Floor - John Vane Science Centre
Charterhouse Square
London
EC1M 6BQ

**Study Statistician:**

Mira Varagunam

Tissue Typing Laboratory
Barts and The London, Queen Mary's School of Medicine and Dentistry
2nd Floor
Medical College Building
Whitechapel
London
E1 1BB

**Other study contacts:**

Professor Marion Macey

Dept of haematology

Royal London Hospital

3^rd^ Floor

80, Newark Street

Whitechapel

London E1 1BB

**IN CASE OF EMERGENCY:**

**Contact Dr Gavin Dreyer**

Department of translational medicine

John Vane Science Building

William Harvey Research Institute

Charterhouse Square

London

EC1M 6BQ

Tel - 07779652081

Gavin.Dreyer@bartsandthelondon.nhs.uk

**Table of Contents**

Study Summary 1

**GLOSSARY OF ABBREVIATIONS …………………………………………………………1**

1. Introduction 2

**1.1 Background 4**

**1.2 Investigational Agent 5**

**1.3 Preclinical Data 6**

**1.4 Clinical Data to Date 6**

**1.5 Dose Rationale and Risk/Benefits 7**

**1.6 Rationale and Risk/Benefits 7**

2 Study Aims and Objectives 7

3 Study Design 8

**3.1 General Design 8**

**3.2 Primary Study Endpoints 8**

**3.3 Secondary Study Endpoints 8**

**3.4 Primary Safety Endpoints 9**

4 Subject Selection and Withdrawal 9

**4.1 Inclusion Criteria 9**

**4.2 Exclusion Criteria 9**

**4.3 Subject SCREENING AND RECRUITMENT 10**

**4.4 Withdrawal of Subjects 11**

**4.4.1 When and How to Withdraw Subjects 11**

**4.4.2 Data Collection and Follow-up for Withdrawn Subjects 11**

5 Study Drug 12

**5.1 Description 12**

**5.2 Product Sourcing Manufacture and Supply 14**

**5.3 Treatment Regimen 14**

**5.4 Method for Assigning Subjects to Treatment Groups 15**

**5.5 Preparation and Administration of Study Drug 15**

**5.6 Subject Compliance Monitoring 15**

**5.7 Prior and Concomitant Therapy 15**

**5.8 Packaging 16**

**5.9 Blinding of Study Drug 16**

**5.10 Receiving, Storage, Dispensing and Return 16**

**5.10.1 Receipt of Drug Supplies 16**

**5.10.2 Storage 17**

**5.10.3 Dispensing of Study Drug 17**

**5.10.4 Return or Destruction of Study Drug 17**

6 Laboratory Assays 17

7 Study Procedures 18

**7.1 Visit 1**

**7.2 Visit 2**

**7.3 Etc.**

8 Statistical Plan 21

**8.1 Sample Size Determination 21**

**8.2 Statistical Methods 22**

**8.3 Subject Population(s) for Analysis**

**8.22 interim analyses**

9 Safety and Adverse Events 23

**9.1 Recording of Adverse Events 23**

**9.2 When Adverse Events are Recorded 25**

**9.3 Study Stopping Rules 25**

**9.4 Unblinding Procedures 25**

**9.5 Medical Monitoring 25**

10 Data Handling and Record Keeping 26

**10.1 Confidentiality 26**

**10.2 Source Documents 26**

**10.3 Case Report Forms 26**

**10.4 Records Retention 26**

11 Study Monitoring, Auditing, and Inspecting 26

**11.1 Study Monitoring Plan 26**

12 Ethical Considerations 27

13 Study Finances 29

**13.1 Funding Source 29**

**13.2 Indemnity for the performance of the study 29**

**13.3 Subject Payments 29**

14 Sponsorship 29

15 Publication Plan 29

16 References 29

17 Attachments 32

**18 Appendix 1 Study ammendments after 1/3/10**

# Study Summary

**Title**

The effect of Vitamin D on the microcirculation of patients with chronic kidney disease (CKD) and vitamin D deficiency

**Protocol Version Number and Date**

Version 1.3, 25/2/10

**Short Title**

Vitamin D, CKD and the microcirculation

**Methodology**

Double blind, randomised controlled trial comparing oral ergocalciferol with placebo in patients with chronic kidney disease and vitamin D deficiency.

**Study Duration**

Each patient studied for 7 months. We anticipate 6 months to complete recruitment. Post study analysis will take approximately 4 months.

See appendix 1 for additional study details

**Study Centres**

The Royal London Hospital, London, UK

**Objectives**

To determine if vitamin D therapy in a cohort of patients with chronic kidney disease and vitamin D deficiency improves microcirculatory function, progression of chronic kidney disease and other key cardiovascular parameters.

**Number of Subjects/Patients**

80 patients (40 in each arm)

15 normal, healthy subjects will also be recruited to determine normal values for the study procedures

**Main Inclusion Criteria**

Inclusion criteria: eGFR between 15 and 60 ml/min/1.73m^2^, vitamin D levels <40nmol/L, no evidence of diabetes mellitus and not receiving either haemodialysis or peritoneal dialysis. Age 18-80

**Statistical Methodology and Analysis**

All data will be analysed by the principal investigator with the support of a trained statistician and the chief investigator. The data will be assessed for normality and the relevant parametric or non−parametric tests applied. The statistical programmes SPSS and STATA will be used.

# Glossary of abbreviations – study specific

| **ADMA - asymmetric dymethyl arginine**  Naturally occurring inhibitor of nitric oxide. Causes vasoconstriction, hypertension and  impaired endothelium dependent vasodilatation. |
| --- |
| **Cardiac MRI – Magnetic resonance imaging**  Detailed imaging of the heart and its surrounding structures. This technique uses no radiation, rather magnetic fields and radio waves generate highly detailed anatomical images |
| **CKD – chronic kidney disease**  A state of chronically impaired kidney function leading to a major increase in cardiovascular morbidity and mortality. It is a global health problem with an increasing incidence and prevalence. |
|  |
| **ECG - elctrocardiogram**  A non-invasive test used to measure heart rate, rhythm and some morphological features  **Echo – echocardiogram**  A non-invasive ultrasound assessment of the structure and function of the hear and surrounding structures |
| **eGFR – estimated Glomerular filtration rate**  A value generated by validated estimation equations which converts serum creatinine into a more functional assessment of kidney function. Expressed as ml/min/1.73m^2^ and usually referred to as a percentage of kidney function. |
| **eNOS – endothelial nitric oxide synthase**  An endothelial based enzyme which converts L-arginine to nitric oxide |
| **ESRD – end stage renal disease**  Occurs when there is a very low glomerular filtration rate. Usually associated with significant symptoms and usually requires intervention with renal replacement therapy such as dialysis or transplantation |
| **Iontophoresis**  A non-invasive test to determine microvascular function in the skin. A very small electric current delivers vasoactive drugs to the dermis and response in the blood vessels is measured by a laser Doppler probe. |
| **Laser Doppler flowmetry**  The non-invasive measurement of red blood cell flux in skin micro-vessels by using laser Doppler probes. |
| **LV mass – left ventricular mass**  Measured mass in grams of the left ventricle. Elevated values are associated with disease states such as hypertension. This can readily be assessed by cardiac MRI |
| **Oxidative stress**  A state of adverse chemistry at a cellular level which can lead to cell dysfunction and death. Mediated by reactive oxygen species |
| **PCR – protein/creatinine ratio**  A measure of protein in the urine reflecting Glomerular injury. More sensitive than urine dipsticks. |
| **PWV – pulse wave velocity**  A non-invasive measure of vascular health. Blood pressure cuffs around the neck and thigh inflate and deflate to measure the speed of single pulse waves. Higher values are found in disease states. |
|  |
| **Skin AF -Skin autofluorescence**  A non-invasive measure of accumulated metabolic end products which correlate to overall vascular health.  **SDF – side stream dark field imaging**  This imaging technique utilizes a novel method of reflectance avoidance in which the illuminated light and reflected light travel via independent pathways. Sub lingual capillaries can be imaged in this way. An analysis of the moving cells in the images permits the quantitative measurement of red blood cell flow in the capillaries.  **Glossary of terms – study non-specific**  **AE** Adverse Event  **AR**  Adverse Reaction  **ASR**  Annual Safety Report  **CI** Chief Investigator  **CRF**  Case Report Form  **JRO**  Joint Research and Development Office  **IMP**  Investigational medicinal product  **NHS R&D** National Health Service Research & Development  **PI** Principle Investigator  **QA**  Quality Assurance  **QC** Quality Control  **RCT** Randomised Control Trial  **REC**  Research Ethics Committee  **SAR** Serious Adverse Reaction  **SAE** Serious Adverse Event  **SOP**  Standard Operating Procedures  **SmPC**  Summary of Product Characteristics  **SSAR** Suspected Serious Adverse Reaction  **Subject**  An individual who takes part in a clinical trial  **SUSAR** Suspected Unexpected Serious Adverse Reaction |
|  |

# Introduction

## Background

Cardiovascular (CVS) diseases are the major cause of death in patients with renal failure, accounting for approximately half of all deaths (1). Patients with CKD are far more likely to die of cardiovascular disease than progress to end stage renal disease (ESRD) (2). Recently, vitamin D deficiency has been identified as a non-traditional CVS risk factor (3,4,5). Currently, vitamin D is not routinely prescribed in the early stages of CKD.

Vitamin D has been shown to improve microcirculatory function in diabetic patients with vitamin D deficiency (6). Microcirculatory dysfunction itself is now considered a marker of cardiovascular health and a predictor of future cardiovascular events (7)

A number of recently published observational studies (8,9,10) provide support to the possible CVS protective role of vitamin D in the ESRD population. These studies describe the effect of vitamin D analogues on all-cause and CVS mortality in patients on haemodialysis, and demonstrated that mortality was inversely related to treatment with vitamin D analogues.

There is a pressing need to examine the effects of vitamin D therapy in the early stages of CKD. Early detection of microcirculatory dysfunction, before ESRD is reached, will provide a powerful tool for predicting future CVS events and thus provide an opportunity to intervene with therapies including vitamin D at an early stage of renal dysfunction. This will determine if important reductions in CVS risk can be achieved at a stage when the vascular system is likely to be more responsive to pharmacological intervention and where the risk of CVS events far outweighs the progression to ESRD.

This trial will establish both the therapeutic benefit of early treatment with vitamin D and determine how the microcirculation responds to vitamin D therapy at a clinical and in vitro level. Potential benefits include the long term reduction of CVS risk by determining the place of vitamin D therapy in early CKD, establishing how microcirculatory assessments can be utilised in clinical practice and further studying the link between endothelial dysfunction and vitamin D deficiency.

## Investigational Agent

Ergocalciferol - Vitamin D2, a fat-soluble vitamin important for many biochemical processes including the absorption and metabolism of calcium and phosphorus. In vivo, ergocalciferol is formed after sun (ultraviolet) irradiation of plant-derived ergosterol, another form of vitamin D occurring naturally in human skin.

Ergocalciferol is used to treat chronic hypocalcemia, hypophosphatemia, rickets, and osteodystrophy associated with various medical conditions including chronic renal failure, familial hypophosphatemia, and hypoparathyroidism (postsurgical or idiopathic, or pseudohypoparathyroidism).

Ergocalciferol is indicated for the prevention and treatment of vitamin D deficiency states. Vitamin D deficiency may occur as a result of inadequate nutrition, intestinal malabsorption, or lack of exposure to sunlight, but does not occur in healthy individuals receiving an adequate balanced diet and exposure to sunlight.

Vitamin D is essential for promoting absorption and utilization of calcium and phosphate from the intestine and for normal calcification of bone. Along with parathyroid hormone and calcitonin, it regulates serum calcium concentrations by increasing serum calcium and phosphate concentrations as needed. Vitamin D stimulates calcium and phosphate absorption from the small intestine and mobilizes calcium from bone.

The molecular weight of ergocalciferol is 396.65. It has a plasma half life of 19 to 48 hours (stored in fat deposits in body for prolonged periods).

A dose of 1.25mg (50,000IU) to 5mg daily has been recommended. The dose should be adjusted according to the severity of the condition. This medicine is delivered by oral administration

Adverse events are generally associated with excessive intake of ergocalciferol leading to the development of hypercalcaemia. The symptoms of hypercalcaemia can include; anorexia, nausea, vomiting, diarrhoea, loss of weight, headache, polyuria, thirst, vertigo, constipation, fatigue, bone pain, muscle weakness, abdominal pain, mental disturbances, impaired renal function, kidney stones and cardiac arrhythmias.

A single acute overdose is virtually non-toxic and requires supportive treatment with liberal fluids only. Treatment of chronic overdose with resulting hypercalcaemia consists of immediate withdrawal of the vitamin, a low calcium diet, and generous fluid intake. Severe cases may require hydration with intravenous saline together with symptomatic and supportive treatment as indicated by the patient's clinical condition. Plasma calcium and U&E's should be monitored.

## Preclinical Data

The endothelium is strategically located between the muscular wall of blood vessels (of all sizes) and the blood stream. It responds to stimuli including pressure, shear stress, and hormonal agents which mediate relaxation and contraction of the underlying vascular muscular wall. These stimuli prompt endothelial cells to release agents that regulate vasomotor function, trigger local inflammatory reactions, and affect homeostasis (11).

Endothelial dysfunction results in reduced vasodilation, a pro-inflammatory state, and pro-thrombotic consequences that predispose to artherosclerotic plaque formation which in turn is associated with cardiovascular disease processes, including hypertension (12), coronary artery disease (13), chronic heart failure (14), peripheral artery disease (15) and diabetes (15).

Reduced endothelial vasodilatory responses are mainly caused by reduced nitric oxide generation due to down-regulation of eNOS, the enzyme mostly responsible for its generation. This process has been shown to be multi-factorial (16,17,18,19).

Recent unpublished evidence also suggests that vitamin D deficiency can impair the activation of eNOS. A study involving vitamin D receptor knock out mice, where eNOS activity was reduced, showed reversal of endothelial dysfunction by calcium supplementation (20). It has also been shown that exogenous vitamin D prevents platelet activation and platelet-monocyte aggregate formation due to down regulation of the renin-angiotensin system (21,22). It is thought that these processes will accelerate atherosclerotic plaque formation.

However, many important markers of endothelial health and function have not been assessed in the setting of a clinical trial. This study will provide an opportunity to study the endothelium at a cellular level to determine the molecular pathways by which vitamin D therapy mediates its effects.

**1.4 Clinical Data to Date**

It is logical to study the earlier stages of CKD to determine if therapeutic interventions can reduce cardiovascular morbidity and mortality. However, current data regarding vitamin D and CKD is observational (23) and a large, prospective, randomised controlled trial of vitamin D therapy in CKD patients, with particular focus on the microcirculation, has yet to be conducted (24). The status of the microvascular beds of both the heart and kidney are reflected by subdermal capillary function (25,26). However, intricate analysis of the effects on the microcirculation of vitamin D supplementation in a population with CKD has yet to be undertaken. A recent review article (27) has highlighted the need for further assessments of microcirculatory dysfunction in patients with CKD as a method for predicting adverse CVS outcomes.

## 1.5 Dose Rationale and Risk/Benefits

A dose regimen of ergocalciferol 50,000 IU weekly for 12 weeks and then monthly for 3 months has been shown to be effective at both raising serum vitamin D levels and lowering PTH levels in a similar cohort which is the precise effect we hope to achieve in our study group (28). No patient developed the most common side effect of hypercalcaemia and no other adverse effects were reported. A further study (29) of weekly ergocalciferol (50,000 IU/week for 24 weeks) was also shown to raise serum vitamin D levels with no adverse events reported. We have chosen the dose regimen in the K/DOQI guidelines for the replacement of vitamin D in patients with CKD. This consists of 50,000IU of ergocalciferol weekly for 1 month and then monthly for 5 months. As in the studies above, we anticipate a rise in serum vitamin D levels to therapeutic but well below toxic levels.

## 1.6 Rationale and Risk/Benefits

Techniques to assess the microcirculation are quick, non-invasive and reproducible and could become a powerful risk assessment tool available in routine clinical practice. A number of studies assessing vitamin D therapy in CKD are underway (30), but none have undertaken an analysis of microcirculatory dysfunction either in vivo with clinical assessments of microcirculatory function or in vitro by determining the mechanism of action of vitamin D on the endothelium.

This trial offers a unique opportunity to evaluate the place of vitamin D therapy in early CKD. By furthering our knowledge of the in vitro and in vivo effects of vitamin D therapy in patients with CKD, we will have the opportunity to enhance current practice, further reduce CVS risk and develop a greater understanding of the interaction between vitamin D and the endothelium.

# Study Aims and Objectives

**Primary study objective** – to evaluate the effects of vitamin D on the microcirculation of patients with chronic kidney disease (CKD) and vitamin D deficiency.

**Secondary study objective** – to evaluate the effects of vitamin D on key clinical parameters in patients with chronic kidney disease (CKD) and vitamin D deficiency.

# Study Design

## General Design

Single centre, double blind, randomized control trial of oral ergocalciferol compared to placebo.

## Primary Study Endpoints

**1. Microcirculatory studies** – pulse wave velocity (PWV), iontophoresis of acetyl choline (AcH) and sodium nitroprusside (SNip), skin autofluoresence (AF) and side stream dark field imaging of the sub lingual microcirculation.

Pulse wave velocity – we expect PWV to decrease after vitamin D therapy.

Iontophoresis – we expect lower skin micro-vessel resistance as measured by laser Doppler Flowmetry (LDF) after vitamin D therapy.

Skin autofluorescence – we expect lower deposition of advanced glycation end products after vitamin D therapy.

Sublingual microvascular flow using sidestream darkfield (SDF) imaging – we expect enhanced flow in patients treated with vitamin D

## Secondary Study Endpoints

**1. Quality of life** – we expect improved quality of life in the treatment group as measured by the SF 36 questionnaire.

**2.** A range of in vitro assessments to establish measures of oxidative stress and platelet activity will be conducted in a laboratory at the Royal London Hospital with an established protocol for these techniques.

**3. Left ventricular mass** – we expect reduced LV mass in the treatment group as measured by cardiac magnetic resonance imaging (MRI).

**4. Carotid artery intimal thickness** – we expect unchanged intimal thickness in the treatment group as assessed by ultrasound of the carotid arteries. This will imply that any enhancement to vascular function occurs as a result of changes to the function rather than structure of the conduit vessels.

**5. Blood pressure** – we expect blood pressure to reduce over the study duration in the treatment group as measured by 24 hour ambulatory blood pressure.

**6. Cardiac events** – we will record any cardiac events (myocardial infarction, stroke etc) that occur in either group

**7.** **Progression of kidney disease** – we expect the treatment group to progress slower as assessed by estimated glomerular filtration rate (eGFR) at 0 and 6 months

**8.** **Proteinuria** – we expect reduced proteinuria in the treatment group as assessed by urine protein:creatinine ratio (PCR)

## Primary Safety Endpoints

1. Clinically significant Hypercalceamia

This is the most likely safety issue to occur during the trial. Previous studies (28,29) have demonstrated that our proposed dose regimen is very unlikely to cause hypercalcaemia while providing an effective increase in vitamin D levels. Furthermore, it has been reported that vitamin D supplementation as per current guidelines is extremely unlikely to cause hypervitaminosis D and that the toxic effects of vitamin D (hypercalcemia) only start to occur at levels > 200 nmol/L, well above the expected vitamin D level for the dose regimen proposed in this study (31,32).

# Subject Selection and Withdrawal

## Inclusion Criteria

1. eGFR between 15 and 60 ml/min/1.73m2
2. Serum 25 (OH) vitamin D levels <40nmol/L as measured within the last 2 months
3. No evidence of diabetes mellitus (fasting blood sugar <7.1, not taking any diabetic medication)
4. Not receiving haemo or peritoneal dialysis
5. No dialysis therapy within the last 3 months
6. Age > 18 years and < 80 years
7. Patient agrees not use any medications (prescribed or over-the-counter including herbal remedies) judged to be clinically significant by the Principal Investigator during the course of the study.
8. Able to understand and sign the written Informed Consent Form.
9. Able and willing to follow the Protocol requirements.

## Exclusion Criteria

# 1. Currently receiving oral ergocalciferol at any dose

# 2. Received IM ergocalciferol therapy within last 3 months

# 3. Receiving renal replacement therapy of any type or having recently received any form of dialysis (within 3 months)

# 4. Pacemaker or any other implanted cardiac device

# 5. Serum calcium above 2.6 mmol/L at screening

# 6. Pregnant or lactating

# 7. Known hypersensitivity to ergocalciferol

8. Patient known to have a condition which predisposes to hypercalcaemia (multiple myeloma, sarcoidosis, other granulomatous disease)

9. Initial blood pressure of >160/100 mmHg

10. History of significant liver disease or cirrhosis

11. Anticipated requirement for dialysis in 6 months

12. Malabsorption, severe chronic diarrhea, or ileostomy

13. Known diagnosis of hypervitaminosis D

14. Known to have diabetes mellitus

15. Known to have renal calculi

16. Known to have systemic sclerosis, Raynaud’s phenomenon or other disease associated with known microcirculatory dysfunction

17. Concurrent participation in any other research study

18. Unwilling or unable to complete study protocol

### Females of childbearing potential and males must be willing to use an effective method of contraception (hormonal or barrier method of birth control; abstinence) from the time consent is signed until 6 weeks after treatment discontinuation.

### Females of childbearing potential must have a negative pregnancy test within 7 days prior to being registered for protocol therapy.

### NOTE: Subjects are considered not of child bearing potential if they are surgically sterile (they have undergone a hysterectomy, bilateral tubal ligation, or bilateral oophorectomy) or they are postmenopausal.

## Subject Recruitment and Screening

The examinations will be carried out in a quiet, stable, draught free environment, Temperature and humidity will be controlled (21±1^o^ C, relative humidity 30-40%). Patients will be instructed to have a light breakfast, avoiding fatty foods, tobacco and caffeine and will be asked to abstain from vigorous exercise from the previous evening onwards. They will be supine for 10 mins during acclimatisation.

**Healthy subjects:**

Healthy volunteers will be recruited by advertisement to staff and students of Queen Mary, University of London and Barts & the London NHS Trust and to the general community, as approved by a National Research Ethics Service (NRES) approved Research Ethics Committee.

Fifteen (15) normal, healthy subjects will be recruited to undergo two microcirculatory assessments to determine reproducibility, coefficient of variance and normal ranges for the assessments described in section 7 (iontophoresis, skin autofluorescence, side stream dark field imaging and pulse wave velocity). They will not receive any pharmacological preparation. A blood test will be taken for both baseline and specialist laboratory assays as described in section 6. Patients will be asked to provide written, informed consent before participating.

**Subjects with CKD:**

Subjects will be recruited by an investigator from general nephrology outpatient clinics at the Royal London Hospital. Patients will be contacted after a clinic visit if they meet inclusion/exclusion criteria and offered the chance to participate in the study. Female patients will be asked to undergo a pregnancy test at the time of the initial screening.

Patients will be provided with a full explanation of the nature, purpose and requirements of the study including Patient Information Sheets and Consent Forms. They will be invited to participate in a screening evaluation, which will include a medical history and physical examination. The subject’s General Practitioners will be informed of an individual’s agreement to participate. Results of the screening evaluation will determine eligibility for entry into the study. Patients will have the opportunity to discuss the trial further with an investigator before giving consent.

## Withdrawal of Subjects

### When and How to Withdraw Subjects

Withdrawal to occur if:

1. Pregnancy occurs during the trial (the CVS effects of pregnancy are likely to significantly alter the assessments of the microcirculation and therefore will invalidate study results)

2. Episode of symptomatic hypercalcaemia

3. Adverse reaction to any of the study drugs or procedures as judged by the principal investigator.

4. Patient request to withdraw for any reason

5. Permanent pace maker or other implantable cardiac device fitted during trial

6. Patients receive dialysis therapy during trial duration

7. Patients undergo kidney transplantation

8. Patients develop diabetes mellitus during trial duration

9. Failure to comply with study protocol, as judged by the principal investigator

- Intercurrent medical events and prescription of new medication by the patient’s general practitioner that are judged by the principal investigator not to interfere with the study protocol will not result in withdrawal. Such events will be recorded in the CRF.
- New patients will be recruited to ensure that the study meets the patient numbers in the power calculation
- If patients choose to withdraw or are withdrawn due to the criteria listed in 4.4.1 prior to completion of the study, they will be replaced unless they have withdrawn due to confirmed study drug toxicity.
- All patients who withdraw will be offered an appointment with their usual nephrologist after withdrawal to provide ongoing clinical care
- All withdrawals will be documented on the CRF and the patient’s general practitioner and usual nephrologist will be informed.

### Data Collection and Follow-up for Withdrawn Subjects

We will use data and urine/blood samples which have already been collected for study analysis providing the patient gives consent for this to happen. Patients will have the opportunity and choice to inform the trial team how their health has been over the study duration even if they withdraw.

# Study Drug

## Description

Ergocalciferol capsules and matching placebo capsules will be provided by Mawdsley Brooks (UK). Ergocalciferol capsules will contain 50,000 International Unit (IU) (1,25mg) of ergocalciferol. The ergocalciferol capsule will be over-encapsulated into a size 00, red, empty gelatine capsule measuring 20mm and containing no markings. The placebo capsule will physically match the over-encapsulated ergocalciferol exactly.

Ergocalciferol is a white, colourless crystal, insoluble in water, soluble in organic solvents and slightly soluble in vegetable oil. One unit of vitamin D_2_ is equivalent to one IU, and 1 mcg of vitamin D_2_ is equal to 40IU.

Ergocalciferol, also called vitamin D_2_ is 9,10-secoergosta-5,7,10 (19), 22tetraen-3-ol, (3ß,5Z,7E,22E)-: (C_28_H_44_O) with a molecular weight of 396.65.

It has the following molecular composition:


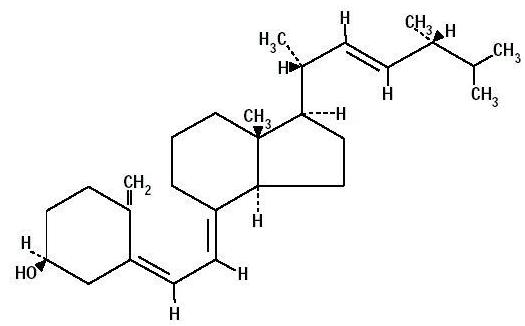


## Clinical pharmacology:

The in vivo synthesis of the major biologically active metabolites of vitamin D occurs in 2 steps. The first hydroxylation takes place in the liver (to 25-hydroxyvitamin D) and the second in the kidneys (1,25 hydroxy vitamin D). Vitamin D metabolites promote the active absorption of calcium and phosphorus from the small intestine, thus elevating the serum calcium and phosphate level sufficiently to permit bone mineralization. Vitamin D metabolites also mobilize calcium and phosphate from bone and probably increase the reabsorption of calcium and perhaps also phosphate from the renal tubules.

There is a lag of 10-24 hours between the administration of vitamin D and the initiation of its action in the body due to the necessity of synthesis of the active metabolites in the liver and kidneys. Parathyroid metabolism is responsible for the regulation of this metabolism in the kidneys.

Indications:

Hypoparathyroidism, refractory rickets, familial hypophosphataemia, vitamin D deficiency

Contraindications:

Hypercalcaemia, malabsorption syndrome, hypervitaminosis D, abnormal sensitivity to the toxic effects of vitamin D

Drug interactions:

Mineral oil interferes with the absorption of fat-soluble vitamins, including vitamin D preparations.

Administration of thiazide diuretics to hypoparathyroid patients who are concurrently being treated with ergocalciferol may cause hypercalcaemia.

Carcinogenesis, mutagenesis and impairment of fertility:

No long term animal data is available

Pregnancy:

Animal studies have shown fetal abnormalities in several species associated with hypervitaminosis D which result in similar findings of supravalvular aortic stenosis described by Black in 1963.

For the protection of the fetus, the use of vitamin D in excess of the daily recommended amount during normal pregnancy should be avoided unless in the judgement of the physician, the potential benefits outweigh the risks.

Nursing mothers:

Caution should be exercised when ergocalciferol is administered to a nursing mother as there is one report of hypercalcaemia in an infant

Adverse events are generally associated with excessive intake of ergocalciferol leading to the development of hypercalcaemia. The symptoms of hypercalcaemia can include; anorexia, nausea, vomiting, diarrhoea, loss of weight, headache, polyuria, thirst, vertigo, constipation, fatigue, bone pain, muscle weakness, abdominal pain, mental disturbances, impaired renal function, kidney stones and cardiac arrhythmias.

A single, acute overdose is virtually non-toxic and requires supportive treatment with liberal fluids only. Treatment of chronic overdose with resulting hypercalcaemia consists of immediate withdrawal of the vitamin, a low calcium diet, and generous fluid intake. Severe cases may require hydration with intravenous saline together with symptomatic and supportive treatment as indicated by the patient's clinical condition. Plasma calcium and U&E's should be monitored.

## Product Sourcing Manufacture and Supply

The study drug and placebo will be sourced and supplied by Mawdsley Brooks UK. Manufacture of ergocalciferol is by Sanofi-Aventis Pharmaceuticals, 300, Somerset corporate boulevard, Bridgewater NJ 08807-2454 USA. Sanofi Aventis hold the Marketing Authorisation and necessary licence for product use. Mawdsley Brooks UK are supplied with ergocalciferol from the manufacturer by Pharmaceuticals Trade Services inc, PO Box 561 Gaitier, MS 39553 USA. Manufacture of the placebo is by

Ipswich Hospital NHS Trust Pharmacy Manufacturing Unit, Heath Road, Ipswich, UK.

## Treatment Regimen

The replacement regimen is based on current guidelines for vitamin D replacement in CKD (33)

After randomization at visit 3, patients will take 1 capsule of study drug (either placebo or active ergocalciferol) weekly for four weeks and then monthly for the following 5 months (9 doses in total).

Study visit 3: After randomization, 9 capsules of study medication will be dispensed at this visit and the patient will be observed swallowing the first dose of medication. . Patients will be told to bring back the study medicine at the next and all subsequent visits.

Study visit 4: the number of capsules remaining will be counted and the patient will be observed swallowing the medication.

Study visit 5: the number of capsules remaining will be counted and the patient will be observed swallowing the medication.

Study visit 6: the number of capsules remaining will be counted and the patient will be observed swallowing the medication.

Study visit 7: the number of capsules remaining will be counted and the patient will be observed swallowing the medication.

Study visit 8: the number of capsules remaining will be counted and the patient will be observed swallowing the medication. This will be the last dose of study medication.

Study visit 9 (last visit): no medication will be taken.

No further study drug will be dispensed after visit 3 unless the patient has lost or mislaid the medication and has an insufficient supply to complete the trial

## Method for Assigning Subjects to Treatment Groups

Subjects will be randomised to either ergocalciferol or placebo in a 1:1 fashion using a random number generator.

## Preparation and Administration of Study Drug

All study medication will be prepared and logged in the Royal London Pharmacy department. Medication will be dispensed by trained pharmacy personnel or an investigator. Subjects will be observed swallowing the medication at visits 3, 4, 5, 6, 7 and 8 by either pharmacy staff or an investigator.

## Subject Compliance Monitoring

During the first month of the trial, patients will receive nine capsules of study drug. These will be provided in a sealed container which will be checked at subsequent study visits to ensure compliance. This will avoid over frequent visits to the trial clinic and will maximize compliance. At subsequent study visits, which require attendance at the hospital, patients will be observed swallowing their medication to ensure compliance. To enhance compliance, a text message will be sent to patients if they have a mobile phone (and if they have indicated they are happy to receive a message from the study team) on the day they are due to take the study medication. The message will read “Dear NAME this is a reminder to take your vitamin D study medication today. Thank you”

## Prior and Concomitant Therapies

Prior therapies:

There will be a washout period of 2 weeks for all vitamin D therapies currently being taken by participants. The most commonly prescribed oral vitamin D analogues all have a half life of less than 14 days (Alfacalcidol— 3 hours, Calcitriol—3 to 6 hours, Ergocalciferol—19 to 48 hours) (34). Participants will be screened for use of all over the counter medicines which may contain vitamin D. It is very unlikely patients will suffer any side effects from stopping these medications for this period. Patients will be reassured about the safety of drug withdrawal and the safety and validity of undertaking a randomized, placebo controlled trial. Given that routine supplementation with vitamin D in early CKD is not routine, we anticipate that very few individuals will actually need to stop vitamin D analogues.

The patient’s general practitioner will be contacted to ensure no medicines that may contain vitamin D are being taken by participants without their prior knowledge and that no depot vitamin D preparations have been dispensed in the last 6 months.

Concomitant therapies:

Blood pressure medication should be unchanged for study period but additional therapies can be added at the discretion of the investigators if blood pressure requires clinical intervention on safety grounds. General practitioners will be asked to maintain BP medications at a stable dose. Any changes in medication by the study team will be communicated to GP’s in writing and included in the CRF. Patients will be asked to take any concomitant therapies which can affect microvascular function after clinic visits at which these assessments are made (visits 2,3,4,6,9)

Drugs which are likely to have these particular effects include aspirin and vasodilating anti-hypertensives such as calcium channel blockers. There is no evidence to suggest that iontophoresis of acetylcholine (Ach) and sodium nitroprusside (SNip) is directly affected by the presence of any antihypertensive drugs. However, there is a theoretical chance that there may be some affect on vasodilatiation particularly by drugs with a known vasodilatory effect eg calcium channel blockers. Hence, patients will be asked to take all their daily medications after all microcirculatory assessments have been made.

Aspirin has been shown to affect microcirculatory response to AcH and SNip but this effect can be corrected by measuring skin resistance (35). However, another group has shown no effect of aspirin on the response to AcH (36). The maximum half life of aspirin is 20 hours at high doses but is as low as 6 hours when lower doses are used (34) and therefore even a dose taken the day before iontophoresis assessments is very unlikely to affect the results.

Patients will be asked at each visit if they have been prescribed or if they have bought any other medicinal or herbal product which may interfere with the study. If a new drug is being taken, a decision to withdraw the subject will be made by the Principal Investigator based on its pharmacology and pharmacokinetics. All details of concomitant medications will be recorded in the Case Report Form (CRF).

There are no dietary restrictions or restrictions on the use of tobacco or alcohol during this study but patients will be asked to refrain from smoking for 4 hours before undergoing microcirculatory assessments.

## Packaging

The packaging and labelling of the IMP will be in accordance with applicable local regulatory requirements.

Study medicine kept at the pharmacy in the Royal London Hospital will be in sealed containers with appropriate clinical trial labelling. Each container will contain nine (9) capsules. Patients will receive a labelled container of nine (9) capsules to take home with them after study visit three. These will be labelled in accordance with trial protocol.

## Blinding of Study Drug

Both the investigators and patients will be blinded to the study drug. Mawdsley Brooks UK will facilitate randomisation and blinding of the study subjects. A master randomisation list will be held at Mawdsley Brooks UK with a copy kept at the Royal London Hospital Pharmacy department.

## Receiving, Storage, Dispensing and Return

### Receipt of Drug Supplies

The investigator is responsible for ensuring IMP accountability, including reconciliation of IMP and maintenance of IMP records, throughout the trial in accordance with regulatory requirements.  Upon receipt of IMP, the investigator (or Pharmacist) will check for accurate delivery and acknowledge receipt and signing (or initialling) and dating the documentation provided by the sponsor and returning it to the sponsor.  A copy will be retained for the investigator file.

### Storage

The IMP should be stored at room temperature (25^o^C**/**77^o^F ) in its original container.  On site, all IMP should be stored in a secure location, in a temperature controlled environment, preferably with a temperature log maintained daily, and may be dispensed only by the investigator or by a member of staff specifically authorised by the investigator, or by the pharmacist, as appropriate.  Any deviations from the recommended storage conditions should immediately be reported to the sponsor and the use of the IMP interrupted until the Sponsor has given authorisation for its use.

### Dispensing of Study Drug

The dispensing of the IMP will be carefully recorded on the appropriate drug accountability forms provided by the sponsor, and accurate accounting will be available for verification by the sponsor, and the sponsors monitor at each visit. Information recorded will include:

Dates, quantities, batch numbers, kit numbers for IMP, expiry dates and trial numbers assigned to the subjects.

### Return or Destruction of Study Drug

Any unused IMP must not be discarded or used for any purpose other than the present trial. Subjects should be instructed to return any unused IMP and all empty blisters and packaging.

**In summary the IMP accountability records will include:**

Confirmation of the IMP delivery to the trial site

The inventory at the site of IMP provided by the Sponsor

The use of each dose by each subject

Any returns or unused product

Dates, quantities, batch numbers, kit numbers for IMP, expiry dates and trial numbers assigned to the subjects.

# Laboratory Assays

**Routine blood tests** (to be undertaken after an 8 hour fast):

If a test result from the haematology, biochemistry, or urinalysis falls outside the normal range, the clinical significance of such a result will be evaluated and the need for repeat testing considered by an Investigator.

The following tests will be taken at each clinic visit as specified in the protocol (section 7)

FBC (full blood count), U+E’s (serum electrolytes, urea and creatinine), Ca (calcium), PO_4_ (phosphate), bilirubin, ALT (alanine transaminase), ALP (alkaline phosphatase), CRP (C reactive protein), lipid profile, albumin, glucose, serum magnesium, 25 (OH) vitamin D (vitamin D levels) and PTH

**Other blood tests**

These tests will be taken at visits 3 and 9

1,25 OH vitamin D, Trop T (troponin T), renin, aldosterone

**Routine samples (urine ~ 25 mls):** urine sodium, urine PCR (protein:creatinine ratio)

A range of in vitro assessments to establish measures of oxidative stress and platelet activity will be conducted in a laboratory at the Royal London Hospital with an established protocol for these techniques.

# Study Procedures and Schedule of Assessments

Patients will be examined and assessed by an investigator at the renal outpatient department of the Royal London Hospital. Cardiac MRI will take place at the London Chest Hospital (aprox 20 min walk from RLH)

All assessments will be similar between the two groups. At each study point, participants will undergo the relevant assessment and will also receive one dose of the study drug to ensure compliance.

The table below details each study time point and exactly what will occur. Each Action is explained in more detail below.

| **Visit** | **Drug** | **Action** | **Action** | **Action** | **Action** | **Action** | **Action** | **Action** | **Action** | **Action** |
| --- | --- | --- | --- | --- | --- | --- | --- | --- | --- | --- |
| 0 weeks | Washout |  |  |  |  |  |  |  | Bloods | Examination |
| 2 weeks | n |  | Microcirc |  |  |  |  |  |  | Examination |
| 1 month (4 weeks) | y | ECG | Microcirc | SF 36 | Urine | MRI/echo | 24hr BP | USS neck | Bloods | Examination |
| 2 month | y |  | Microcirc |  | Urine |  |  |  | Bloods | Examination |
| 3 months | y |  |  |  |  |  |  |  | Bloods | Examination |
| 4 months | y |  | Microcirc |  | Urine |  |  |  | Bloods | Examination |
| 5 months | y |  |  |  |  |  |  |  | Bloods | Examination |
| 6 months | y |  |  |  |  |  |  |  | Bloods | Examination |
| 7 months | n | ECG | Microcirc | SF 36 | Urine | MRI/echo | 24hr BP | USS neck | Bloods | Examination |

**Time** – 0 weeks refers to recruitment, consent and enrolment. Randomisation will occur at month 1 (4 weeks after recruitment). The study will end at month 7 after recruitment. Patients will receive the study drug for 6 months in total.

**Drug** – at month 1 (week 4) patients will be randomized to and receive at that visit either placebo or ergocalciferol. Patients will receive all 9 capsules of study medication at this time. They will be required to take 1 dose weekly at home in the first month. Further study medication will be taken at subsequent clinic visits and patients will be observed swallowing their medication either by pharmacy staff or an investigator.

**ECG** – a 12 lead cardiac ECG will be undertaken. This is a painless, non-invasive procedure.

Duration 5 minutes.

**Microcirc** – This process involves four assessments of the microcirculation and will take place at a clinic visit in the same room.

1. Pulse wave velocity. An assessment of the speed of the pulse transmission in the major blood vessels of the body. Patients will have blood pressure cuffs placed around the thigh and neck. The cuff around the neck poses NO DANGER TO THE PATIENT AS IT ONLY INFLATES TO 60mm Hg – BELOW SYSTOLIC BLOOD PRESSURE. At worst, patients may feel a gentle pressure sensation around the neck. FULL RESUCITATION EQUIPMENT IS AVAILABLE ON SITE AND THE INVESTIGATORS AND CLINIC STAFF ARE TRAINED IN ADVANCED LIFE SUPPORT TECHNIQUES

Duration – 5 minutes

2. Iontophoresis. An assessment of skin microvascular function. Two compounds (AcH and SNip) are transmitted through the skin by the use of a very low electrical current into the small blood vessels located just under the skin. A laser Doppler probe records the response of the microvasculature. This is a non-invasive test and the drug dose is too low to cause any systemic absorption. Minor skin irritation has been reported but is usually mild and self limiting.

Duration - 15 minutes

3. Skin autofluorescence – An assessment of the build up of advanced glycation end products (AGE), which are a marker of oxidative stress. Patients place their arm over an ultraviolet light and results of AGE product accumulation are downloaded to a computer. This is a painless and non-invasive test.

Duration – 5 minutes.

Sublingual microvascular flow using sidestream darkfield (SDF) imaging – this is a non-invasive imaging tool to assess intra-vital capillary blood flow. A small camera (0.7cm) with a replaceable sterile cap is placed under the tongue in a similar way to temperature measurement with a mercury thermometer. 3 separate images are collected during one reading. These recordings will occur at visit 3, 6 and 9.

Duration – 5 minutes

**SF 36 –** Patients will be asked to fill in this standardized questionnaire about quality of life.

Duration - 10 minutes.

**Urine –** patients will be asked to give a clean catch urine specimen when they arrive at clinic which will be divided into 3 samples (urine sodium, urine calcium and urine PCR). Urine samples will be spun down so no cellular component will be retained or studied.

Duration – 5 minutes

**MRI –** Cardiac MRI scanning will take place at the London Chest Hospital and will be supervised by specialist medical and radiography staff. We will measure left ventricular mass and ejection fraction. The examination will not usually require contrast media to be injected.

Duration – 1 hour

**Echo –** cardiac ultrasound conducted at the Royal London Hospital

Duration – 15 minutes

**24 hour blood pressure –** Patients will be fitted with a blood pressure cuff and portable recording device which will measure ambulatory blood pressure over a 24 period.

Duration - 24 hours

**Ultrasound neck –** to be undertaken by trained ultrasonographers and an investigator who will be fully trained in this technique. It will involve a gentle, non-invasive scan of the large blood vessels in the neck.

Duration – 10 minutes

**Bloods** – Will be taken by an investigator or trained clinic staff. Only one venepuncture will be required to minimize discomfort.

Duration - 5 minutes

**Examination** – Patients will undergo a routine clinical examination by an investigator including blood pressure measurement

Duration - 10 minutes.

**Recording of study data** – At each visit, clinical entries will be recorded in patient’s record. Data derived from the above investigation will be documented on the case report form. These files will be kept in a locked cabinet in a locked room in the Renal Out Patient department, Royal London Hospital. This department has a lock which requires access via staff ID cards.

**Assessments for efficacy** –

In the ergocalciferol treated group:

Vitamin D levels

We expect these will increase in patients treated with ergocalciferol

Microcirculatory assessments:

We expect pulse wave velocity to reduce, iontophoresis to show improved microcirculatory function by laser colour Doppler, skin AGE deposition to decrease and sub lingual microcirculation to improve.

Imaging

We expect reduction in LV mass and improvement in ejection fraction

Clinical

We expect reduction in BP, progression of renal dysfunction and reduction in proteinuria

**Assessment for safety –** the most likely side effect of ergocalciferol is hypercalcaemia. We will measure calcium levels at each clinic visit and screen patients for the symptoms of hypercalcaemia. If symptomatic hypercalcaemia is present, the patient will be withdrawn from the study as per section 4.4.1. If the serum calcium is elevated in the absence of associated symptoms, as per standard clinical medical care, the study drug will be omitted at that visit. The patient will then attend the next visit as per the protocol and if the calcium level at that visit is below 2.6 mmol/L, the study drug will be administered and the patient will follow the protocol from that point onwards.

# 7. Statistical Plan

## Sample Size Determination

This study is powered to detect a difference in microcirculatory function as assessed by iontophoresis. It is likely that if a difference is seen in microcirculatory function as assessed by iontophoresis that the other microcirculatory assessments will follow the same trend

We expect 85% failure in the placebo group and 50% failure in the intervention arm (35% difference in success). We have set alpha=0.05 and beta=0.1 which gives us 90% power.

Therefore:

n=[((15x85)+(50x50))/35^2]x10.5=32 patients in each arm

In order to ensure we achieve a total of 32 patients in each arm for an adequately powered study, we will recruit 40 patients per arm to allow for withdrawals and protocol violations.

## Statistical Methods

Full statistical support will be provided for data analysis. Differences between means will be analysed by student’s t test. Multiple regression analyses will be applied to determine which factors are associated with improved microvascular function. STATA software will be used for analysis. Baseline demographics will be recorded for all participants. The study design is in line with CONSORT guidelines.

## Subject Population(s) for Analysis

We will recruit patients from the Royal London Hospital general nephrology outpatient clinics who match inclusion criteria as specified previously. Up to 40 patients in each arm will be recruited (80 in total). Patients will be provided with a full explanation of the purpose and details of the study and will receive a patient information sheet. Written informed consent will be obtained. Patients will undergo a screening evaluation to determine if they meet the study inclusion and exclusion criteria. This will include a medical history and physical examination as well as a review of all relevant existing blood tests.

Healthy volunteers will be recruited by advertisement to staff and students of Queen Mary, University of London and The Barts & the London NHS Trust and to the general community, as approved by a National Research Ethics Service (NRES) approved Research Ethics Committee.

## Informed consent procedures

### It is the responsibility of the Investigator, or a person delegated by the Investigator (the delegation log needs to spell out who is authorised to take consent, only GCP trained individual can take consent) to obtain written informed consent from each subject prior to participation in this study, following adequate explanation of the aims, methods, anticipated benefits and potential hazards of the study.

### Ample time must be given for consideration by the patient before taking part. The PI must record when the patient information leaflet (PIL) has been given to the patient. [If the amount of time between the PIL being given and the date of consent is less than 24 hours, the PI needs to explain why this is the case in this study].

### The Investigator or designee must explain the subjects are completely free to refuse to enter the study or to withdraw at any time during the study, for any reason.

### If new safety information results in significant changes in the risk/benefit assessment, the consent form should be reviewed and updated if necessary. All subjects, including those already being treated, should be informed of the new information, giving a copy of the revised form and give their consent to continue in the study.

# Safety and Adverse Events

## Expected Adverse Events

1. Hypercalcaemia

2. Starting any form of renal replacement therapy (haemodialysis, peritoneal dialysis, kidney transplant)

3. Mild skin erythema over the site of the iontophoresis ion chambers

4. Undergoing surgery for dialysis access

5. Hospital admission due to sepsis

6. Admission to hospital due to poorly controlled blood pressure

7. Hospital admission for any form of cardiovascular event eg stroke, myocardial infarction etc.

7. Claustrophobia in MRI scanner

8. Mild skin reaction to ECG labels

9. Any elective admission to hospital which is unrelated to the study protocol or admission to hospital as a result of an unrelated medical procedure

10. Allergic reaction or any other medical problems related to contrast media from the cardiac MRI scan.

## Definition of Adverse Events

Adverse Event

An AE is any untoward medical occurrence in a subject to whom a medicinal product has been administered, including occurrences which are not necessarily caused by or related to that product. An AE can therefore be any unfavourable and unintended sign (including an abnormal laboratory finding), symptom or disease temporarily associated with the use of an Investigational Medicinal Product (IMP), whether or not considered related to the IMP. All such events during this trial will be recorded in the CRF.

Adverse Reaction (AR)

An AR is any untoward and unintended response in a subject to an Investigational Medicinal Product (IMP), which is related to any dose administered to that subject. All adverse events judged by either the reporting investigator or the Sponsor as having a reasonable causal relationship to a medicinal product qualify as adverse reactions. The expression reasonable causal relationship means to convey in general that there is evidence or argument to suggest a causal relationship.

Serious Adverse Event (SAE)

An SAE fulfils at least one of the following criteria:

- Is fatal – results in death (NOTE: death is an outcome, not an event)
- Is life-threatening
- Requires inpatient hospitalisation or prolongation of existing hospitalization
- Results in persistent or significant disability/incapacity
- Is a congenital anomaly/birth defect

## The above is a broad definition of an SAE. Hospitalisations for elective

## procedures and certain expected adverse events may be exempt from

## this reporting process, if specified in the trial protocol. Additional

## significant medical events may be classed as SAEs.

Suspected Serious Adverse Reaction (SSAR)

An SSAR is an adverse reaction that is classed as serious and which is consistent with the information about the medicinal product as set out in the Summary of Product Characteristics (SmPC) or Investigator’s Brochure (IB) for that product.

Suspected Unexpected Serious Adverse Reaction (SUSAR)

The definition of a SUSAR is any suspected unexpected adverse reaction related to an IMP that is both unexpected and serious. In this case the event is not outlined in the Summary of Product Characteristics (SmPC) or Investigator’s Brochure (IB) for that product.

Critical Adverse Events

A critical adverse event is an event which may not be classified as serious but is considered to be important to the evaluation of safety. These events may become apparent as the trial progresses and requires close communication between the sponsor and investigators.

## Recording of Adverse Events

All events will be recorded on the Adverse Event forms in the patient’s record and a copy will be kept in the CRF.

## Notification and Reporting of Serious Adverse Events/SUSAR

**For UK MAI IMP licensed IMPs only:** As the IMP’s used in this project are licensed in the UK and used within their marketing authorization, the EXPECTED SARs (outlined in the SmPCs) will be RECORDED in the subjects notes and in the CRF. No SAE forms will be completed and sent to the sponsor.

UNEXPECTED Serious Adverse Event (SAE’s) will be recorded in the subjects notes, the CRF and in the sponsor SAE form and reported to the JRO within one working day of the PI or co-investigators becoming aware of the event. The co-investigators listed in this protocol will be authorized to sign the SAE forms in the absence of the PI. Suspected Unexpected Serious Adverse Reactions (SUSAR’s) during the trial will be reported to the JRO and the main REC within one working day of the PI or co-investigator becoming aware of the event.

## When Adverse Events are Recorded

Events will be recorded at each patient visit. Patient’s will asked specifically if any expected adverse events have occurred or if any other events have occurred in the interval between the last patient visit. Non-leading questions such “How do you feel?” and “have you had any problems since I last saw you?” will be asked at each visit.

## Study Stopping Rules

Subjects who are withdrawn because of serious adverse events (including a Grade 3 or 4 toxicity (based on the Common Toxicity Criteria (CTC) version 3.0 – included with this document) will not be replaced. The Ethics Committee will be notified in writing of any study withdrawals that may occur as a result of toxicity.

The study will be stopped if there are any major safety concerns involving the patients involved in the trial as raised by the sponsor or the principal investigator. The study will end when the last patient has received the last dose of study medication and undergone the final assessments as in section 7.

## Unblinding Procedures

Patients and investigators will be unblinded if they are admitted to hospital with hypercalcaemia. Unblinding will be required as there are a number of common medical conditions (myeloma, tuberculosis, sarcoidosis etc) that can cause hypercalcaemia and should be ruled out urgently if a study patient who is taking placebo rather than ergocalciferol presents with symptoms/signs of hypercalcaemia.

Mawdsley Brooks UK will hold a master randomisation list which will be able to identify which arm any subject is in. There is a 24 hour trial hotline which can be contacted by clinical staff at any time should unblinding be required for medical reasons. Furthermore, individual code break envelopes will be available to clinicians at the Royal London Hospital. All renal staff will have access to code break procedures and envelopes which will be locked in a secure office with an entry code on the door.

Code-breaks should only occur when absolutely necessary and beneficial to the patient. In the event of a code break, the sponsor and PI will be informed.

## Medical Monitoring

Patients will be reviewed at each visit by an investigator. They will undergo routine evaluations for standard outpatient care as well as more detailed evaluations as required by the trial protocol. This will include a medical history, examination, blood tests for the trial and others as necessary based on clinical evaluation.

**8.10** The Annual Safety reports (ASR) will be sent by the PI to the sponsor and MHRA (the date of the anniversary is the date on the “notice of acceptance letter” from the MHRA) using the sponsor ASR form. The PI will carry out a risk benefit analysis of the IMPs encompassing all events having arisen on the trial.

**8.11** The Annual progress report will be sent to the main REC (the anniversary date is the date on the MREC “favourable opinion” letter from the MREC) and to the sponsor.

# Data Handling and Record Keeping

## Confidentiality

All computers used will be kept on NHS property and password protected. Each patient’s information will be recorded against an individual code which will be stored on a NHS password protected computer. CRF’s will not contain names, rather the study code for that participant and their initials. Every effort will be made to respect patient confidentiality throughout the trial duration.

## Study Documents

All relevant documents and correspondence will be kept in the patient’s records. Computerised trial documents will be password protected and held on NHS computers at the Royal London Hospital. The investigator and sponsor will have access to these records.

## Case Report Forms

The case report from will contain details of the study name, protocol number, subject initials and trial ID code and other relevant information. It will have a check list for the inclusion and exclusion criteria. It will also contain reporting forms for AE’s, SAE’s and SUSAR’s.

CRF’s for all patients, including those excluded from the study for any reason, will be kept and maintained by the PI. CRF forms are to be completed in black pen and in their entirety with no blank spaces allowed unless data is not available or applicable. This fact must be indicated. Corrections must be struck-through and the correct information entered adjacent to this and initialled and dated by the investigator. Completed CRF’s are to be returned to the sponsor as soon as is practical. Copies are to be retained by the Principal investigator.

## Records Retention

All records will be held in the archive system for 20 years following the start of the trial.

# Study Monitoring, Auditing, and Inspecting

## Study Monitoring Plan

The principal and chief investigator will meet after three months to review AE’s, SAE’s and SUSAR’s. The trial may be subject to routine auditing by Research Governance at Barts and the London NHS Trust.

**Phase IV trials:**

It is the CI’s responsibility to ensure that the BLT monitoring template (designed especially for the BLT/QM sponsored CTIMPs) is completed in a CONTINUOUS fashion throughout the study and kept up to date by the co-investigators (for the first part of the report) and by the MONITORS NAMED ON THE FIRST PAGE OF THIS PROTOCOL (for both the first part and the source data verification part of the template.). This trial is a Phase IV trial, using IMPs licensed in the UK and used within their marketing authorisation, this project is therefore risked as a “low risk (risk B)” project and the monitoring report will be sent to the JRO a year after the first consent has been signed and annually there after.

# Ethical Considerations

General considerations:

The study will be conducted in accordance with the principles of the Declaration of Helsinki (1997) (Recommendations guiding Medical Doctors in Biomedical Research Involving Human Subjects).

Every effort will be made to minimize discomfort and disruption to the trial participants.

All relevant study documentation will be submitted to a National Research Ethics Service (NRES) and no trial activity will begin until approval from this body has been received. A copy of ethical approval will be forwarded to the sponsor. If ethical approval is suspended or terminated, the sponsor will be informed immediately. Trial progress will be reported to the ethics committee once a year. All SAE’s will be reported to the ethics committee as soon as possible and at least within 72 hours.

Study specific ethical considerations:

1. The endpoints of this trial (both in vivo and in vitro) have never been addressed in the setting of a randomized clinical trial. New and relevant information which will change practice to enhance patient care and could influence CKD guidelines can be generated by undertaking this study.

2. The study drug, ergocalciferol, is in routine use for vitamin D deficiency in a number of clinical settings. It has a low side effect profile and is considered to be safe and efficacious.

3. Placebo controlled trials have been in routine use in medical research for years. If a benefit is shown in the ergocalciferol arm, patients who have received placebo can be eligible for ergocalciferol treatment when the trial ends.

4. Secondary hyperparathyroidism (SHPT) is a recognized consequence of CKD and is treated by vitamin D therapy. It is possible that patients with modestly elevated PTH levels will receive placebo tablets. This means that their SHPT will go untreated for 6 months. However, an analysis of PTH levels in an existing group of patients at the Royal London Hospital with CKD and vitamin D deficiency has shown a low median PTH value (median 11.7, standard deviation 19.44 pmol/L). This level of PTH is unlikely to have any serious clinical consequences if not treated over the trial duration and we do not believe patients are at risk by potentially not receiving ergocalciferol. Patients will be reassured about the safety of the drug and the safety and validity of undertaking a randomized, placebo controlled trial.

5. Patients will be required to attend more frequently than normal and have more blood tests (with the associated discomfort) than they might expect in their routine care. Other tests will be required which patients may not have had. A full explanation and support will be offered to patients at every stage of the trial. We will undertake as many trial procedures in a single visit as possible to minimize disruption to the patients.

6. Blood and urine samples will be stored for further analysis relating to the trial. Patients will be clearly informed of this.

## Local Regulations/Declaration of Helsinki

I will ensure that this study is conducted in accordance with the Principles of the “Declaration of Helsinki” (as amended in Tokyo (1975), Venice (1983), Hong Kong (1989), South Africa (1996)). <http://www.wma.net/e/policy/b3.htm> or with the laws of the country in which the research is conducted, whichever accords greater protection to the individual. The study must fully adhere to the principles outlined in the Guidelines for Good Clinical Practice” ICH Tripartite Guideline (January 1997)

## Informed Consent (any special conditions ie emergency situations?)

All subjects will provide written informed consent before enrolling in this trial. A full explanation of the study aims and procedures will be given in writing and explained face-to-face by the chief investigator. Patients will have at least 24 hours to decide on their participation. It will be made clear that patients can refuse to participate and withdraw from the trial at any stage for any reason. If any new information becomes available about any of the study drugs or techniques, patients will be informed and if this information changes the risk /benefit profile of the trial, a new consent form will be issued and informed consent will be obtained. Patients will be informed that the trial investigators may need to release clinical details of a patient to other health care professionals in an emergency situation relating to the trial. The date of informed consent will be documented in the CRF. The patients will be given a copy of the consent form.

## Independent Ethics Committee

All relevant information and forms will be submitted to an external ethics committee by the investigators.

This protocol and the accompanying material given to a potential patient (Patient Information Sheet, Consent form and GP letter) as well as any advertising material will be submitted by the Investigator to an Independent Ethics Committee in the UK. Full approval by the Committee will be obtained prior to starting the study and will be fully documented by letter to the Chief Investigator naming the study site, local PI (who may also be the Chief Investigator) and date the Committee deemed the study as permissible at that site.

# Study Finances

## Funding Source

All costs associated with this study will be covered wholly by:

Barts and the London Trust

Royal London Hospital

Whitechapel Road

London

E1 1BB

Contact: Dr Alistair Chesser, Department head

## Indemnity for the performance of the study

This trial will be covered by standard NHS negligence idemnity

## Subject Payments

Subjects will not receive payment for this trial

# Sponsorship

Barts and the London NHS trust, Whitechapel Road, London E1 1BB

# Publication Plan

The trial results will be published in a peer reviewed journal on completion of the study. Manuscripts for publication (abstract and full text articles) will be reviewed by the principal investigator and be made available to the Sponsor for review prior to submission.

# References

1. Locatelli F, Pozzoni P, Tentori F et al. Epidemiology of cardiovascular risk in patients with chronic kidney disease. Nephrol Dial Transplant 2003; 18 [Suppl 7]: vii2–vii9

2. Feehally J, Griffith K E, Lamb E J et al. **Early detection of chronic kidney disease** BMJ 2008;337: **845-847**

3. YC Li, Kong J, Wei M et al. 1,25-Dihydroxyvitamin D(3) is a negative endocrine regulator of the renin-angiotensin system. J Clin Invest 2002; 110: 229–238

4. Muller K, Haahr PM, Diamant M et al. 1,25 Dihydroxyvitamin D3 inhibits cytokine production by human blood monocytes at the post-transcriptional level. Cytokine 1992 4: 506– 512

5. de Boer IH, Ioannou GN, Kestenbaum B et al. 25-Hydroxyvitamin D levels and albuminuria in the Third National Health and Nutrition Examination Survey (NHANES III). Am J Kidney Dis 2007; 50: 69–77

6. Sugden J, Davies J, Witham M et al. Vitamin D improves endothelial function in patients with Type 2 diabetes mellitus and low vitamin D levels. Diabetic Medicine 2008; 25; 320–325

7. Cohn J, Quyyumi A, Hollenberg N, et al. Surrogate Markers for Cardiovascular Disease Functional Markers. Circulatio*n* 2004;109[suppl IV]:IV-31–IV-46

8. Wolf M, Thadhani R. Vitamin D in patients with renal failure: a summary of observational mortality studies and steps moving forward. J Steroid Biochem Mol Biol. 2007;103(3-5):487-90.

9. Tentori F, Hunt WC, Stidley CA et al. Mortality risk among hemodialysis patients receiving different vitamin D analogs. Kidney Int. 2006 Nov;70(10):1858-65.

10. Andress DL. Vitamin D in chronic kidney disease: a systemic role for selective vitamin D receptor activation. Kidney Int. 2006;69(1):33-43.

11. Furchgott RF, Zawadzki JV. The obligatory role of endothelial cells in the relaxation of arterial smooth muscle by acetylcholine. Nature 1980 288: 373–376

12. Schiffrin EL. A critical review of the role of endothelial factors in the pathogenesis of hypertension. J Cardiovasc Pharmacol 2001; 38[Suppl 2]: S3–S6

13. Monnink SH, van Haelst PL, van Boven AJ et al. Endothelial dysfunction in patients with coronary artery disease: A comparison of three frequently reported tests. J Investig Med 2002; 50: 19–24

14. Landmesser U, Spiekermann S, Dikalov S. Vascular oxidative stress and endothelial dysfunction in patients with chronic heart failure: Role of xanthineoxidase and extracellular superoxide dismutase. Circulation 2002 106: 3073–3078

15. Rizzoni D, Porteri E, Guelfi D et al. Structural alterations in subcutaneous small arteries of normotensive and hypertensive patients with non-insulindependent diabetes mellitus. Circulation 2001;103:1238–1244

16. Casar J, Bautista L, Humphries S et al Endothelial nitric oxide synthase genotype and ischaemic heart disease. Circulation 2004; 109: 1359-1365.

17. Landmesser U, Dikalov S, Price SR et al. Oxidation of tetrahydrobiopterin leads to uncoupling of endothelial cell nitric oxide synthase in hypertension. J Clin Invest 2003; 111: 1201–1209

18. Venugopal SK, Devaraj S, Yuhanna et al. Demonstration that C-reactive protein decreases eNOS expression and bioactivity in human aortic endothelial cells. Circulation 2002 106: 1439–1441

19. Kielstein JT, Bode-Boger SM, Frolich JC et al. Asymmetric dimethylarginine, blood pressure, and renal perfusion in elderly subjects. Circulation 2003; 107: 1891–1895

20. Aihara K, Azuma H, Akaike M et al. Disruption of Nuclear Vitamin D Receptor Gene Causes Enhanced Thrombogenicity in Mice. J Biol Chem 2004; 279 (34):35798–35802

21. Ishikawa M, Sekizuka E, Yamaguchi N et al Angiotensin II type 1 receptor signaling contributes to platelet-leukocyte-endothelial cell interactions in the cerebral microvasculature. Am J Physiol Heart Circ Physiol. 2007 292(5):H2306-15.

22. Din JN, Harding SA, Valerio CJ et al. Dietary intervention with oil rich fish reduces platelet-monocyte aggregation in man. Atherosclerosis. 2007; 197: 290-296

23. Shoben A, Rudser K, de Boer I et al. Association of Oral Calcitriol with Improved Survivalin Nondialyzed CKD. J Am Soc Nephrol 2008; 19: 1613–1619

24. Boer I, Kestenbaum B. Vitamin D in chronic kidney disease: is the jury in? Kidney Int 2008; 74: 985-987

25. Pignocchino P, Conte MR, Scarnato S et al. Study of cutaneous microcirculation using the laser-Doppler method in syndrome X. Cardiologia 1994; 39: 193–197

26. Economides PA, Caselli A, Zuo CS et al. Kidney oxygenation during

water diuresis and endothelial function in patients with type 2 diabetes

and subjects at risk to develop diabetes. Metabolism 2004; 53: 222–

227

27. McIntyre C, John G, Jefferies H. Advances in the cardiovascular assessment of patients with chronic kidney disease. NDT Plus 2008; 6: 383–391

28. Al-Aly Z, Qazi R, González E. Changes in Serum 25-Hydroxyvitamin D and Plasma Intact PTH Levels Following Treatment With Ergocalciferol in Patients With CKD. Am J Kidney Dis 2007; 50:59-68.

29. Blair D, Byham-Gray L, Lewis E et al. Prevalence of vitamin D [25(OH)D] deficiency and effects of supplementation with ergocalciferol (vitamin D2) in stage 5 chronic kidney disease patients. J Ren Nutr. 2008;18(4):375-82

30. <http://clinicaltrials.gov/>. Last accessed October 24, 2008

**31. Vieth R.** Why the optimal requirement for Vitamin D3 is probably much higher than what is officially recommended for adults. J Steroid Biochem Mole Biol 2004: 89–90; 575–579

32. Jones G. Pharmacokinetics of vitamin D toxicity. Am J Clin Nutr 2008;88(suppl):582S– 6S

33. K/DOQI Clinical Practice Guidelines for Bone Metabolism and

Disease in Chronic Kidney Disease. *Am J Kid Dis 2003;*  42(4):, Suppl 3 S16.

34. www.drugs.com (last accessed 24/11/08)

35. Ramsay J, Ferrell, W, Geer I et al. Factors Critical to Iontophoretic Assessment of Vascular Reactivity: Implications for Clinical Studies of Endothelial Dysfunction. J Cardiovasc Pharmacol 2002; 39:9–17

36. Morris SJ, Shore AC. Skin blood flow responses to the iontophoresis of acetylcholine and sodium nitroprusside in man: possible mechanisms. J Physiol 1996;496:531–42.

**Attachments**

This section should contain all pertinent documents associated with the management of the study. The following is a list of attachments, those with an astrix* must be submitted to the Ethics Committee with the protocol.


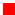
 Consent Form*


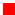
 Information Sheet*


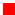
 GP letters / advertisement / any other letters/documents to be given/sent to subjects/patients*


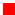
 Layman’s Summary*


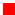
 Peer Review both Internal and External*


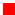
 Investigator Agreement (for any investigator, other than sponsor-investigator, who participates in the study)*

- Study Procedures Flowchart/Table
- Case Report Form
- Core Lab Instructions To Investigators
- Specimen Preparation And Handling (e.g. for any specialized procedures that study team must follow to process a study specimen, and/or prepare it for postage/courier/shipment)
- Drug Conversion Plan (e.g. if there is a special regimen for transitioning a subject from their baseline medication over to study medication)
- Antidote Preparation And Delivery (e.g. special instructions for preparing and delivering any therapy designed to reverse the effects of the study drug, if applicable)
- Study Plan Flowchart
- Source Data Identification List
- Study costing

Standard operating procedures

Pulse wave velocity

1. Subjects will be assessed using the VICORDER - PORTABLE CARDIO-VASCULAR TESTING SMART MEDICAL UK
2. In a quiet, temperature controlled room, patients will be placed at 45 degrees supine on an examination couch
3. PWV measures will be taken from carotid artery to brachial artery and femoral artery using both left and right sided sites
4. Each measure will be repeated twice
5. A tape measure will be used to measure the distance between each BP cuff
6. As per section 7, patients will be asked to refrain from caffeinated beverages or nicotine intake for four hours prior to assessment

Skin autofluorescence

1. Subjects will be assessed using the DiagnOptics’ AGE Reader (Netherlands)
2. This will be connected to a standard personal laptop computer
3. Patients will be assessed in the same environmental conditions (and just after) pulse wave velocity assessment
4. Forearm skin (left and right) will be gently cleaned with an alcohol based swab and any dead skin will be removed by gently placing adhesive medical tape over the affected skin areas.
5. 2 readings will be taken from the left and right forearm

Iontophoresis

1. This will occur immediately after skin autofluorescence under the same environmental conditions
2. Iontophoresis will be conducted and measured by a MIC 2 controller and DRT4 LDF monitor (Moor instruments, UK)
3. Iontophoresis will be assessed in response to 1% AcH and 1% SNip.
4. Forearm skin (left and right) will be gently cleaned with an alcohol based swab and any dead skin will be removed by gently placing adhesive medical tape over the affected skin areas.
5. The following doses will be administered:

| **Current (mA)** | **ACH** | **SNip** |
| --- | --- | --- |
| 0 | 60 sec | 60 sec |
| 10 | 30 sec | 30 sec |
| 0 | 60 sec | 60 sec |
| 20 | 30 sec | 30 sec |
| 0 | 60 sec | 60 sec |
| 35 | 30 sec | 30 sec |
| 0 | 60 sec | 60 sec |
| 50 | 30 sec | 30 sec |
| 0 | 60 sec | 60 sec |
| 75 | 30 sec | 30 sec |
| 0 | 60 sec | 60 sec |

Sub lingual side stream dark field microscopy

1. Several recordings of the sublingual microcirculation will be made each of 60 seconds
2. This will be conducted by a side stream dark field imager (Microvision, UK)
3. Sterile probes will be used for each patient
4. The probe is inserted under the tongue for 60 seconds on 2 occasions 1 minute apart
5. Video images will be recorded for analysis at a later date

Blood samples

1. If required by protocol, venesection will occur under sterile conditions from the dominant arm.

A single sample of 100 mls will be taken at visits 3,4,6,9. At other visits, 20 mls of blood will be taken.

3. Labelling: Tubes will be labelled with the following information

Pt initials

Pt trial number

Date

Time

Protocol number

**4. Residual Samples:** All residual samples will be destroyed following completion of planned assays.

Urine samples

1. As required by the protocol, a ~25 ml mid stream urine sample will be collected

2. Urine samples will be split into two sterile containers. One sample will be analysed at the Royal London Hospital for urine PCR and Na. Another sample will be stored for future analysis in a -80 degree Celsius fridge at the Royal London Hospital. This will be centrifuged so only the acellular component is retained.

**3. Residual Samples:** All residual samples will be destroyed following completion of planned assays.

Other clinical samples

1. Cardiac MRI: To be conducted at the London Chest Hospital

This examination is carried out by specialist doctors and nurses.

The scan lasts approximately 1 hour and will measure left ventricular ejection fraction and left ventricular mass

2. Carotid ultrasound: To be conducted at the Royal London Hospital using a specialist vascular probe and ultrasound machine. 2 readings of carotid intimal thickness will taken from the left and right carotid arteries at visit 3 and 9. Mean values will be recorded. A specialist vascular ultrasound and probe are available. The software has a specific protocol for measuring carotid intimal media thickness.

3. ECG: To be conducted at the Royal London Hospital. Patients will be placed at 45 degrees supine in the same environmental conditions mentioned previously. A standard 12 lead ECG will be recorded.

4. 24 hour BP: This will be conducted as an outpatient on a day to suit the patient. Formal reports will be reviewed and mean BP measures entered in the CRF.

**APPENDIX 1**

**STUDY AMMENDMENTS MADE AFTER 1/3/10**

**Additional study arm:**

**Clinical bacjground**

The investigators will extend the study to include patients with a serum vitamin D level of <70nmol/L. While a value of >70nmol/L has arbitrarily been considered to represent vitamin D sufficiency, to our knowledge, there are no studies of circulatory function in patients with CKD at this level. We believe that the uraemic milieu associated with CKD means that even a vitamin D level of 70nmol/L is insufficient to optimise microvascular function. By providing vitamin D supplementation to this group of patients, we will have a unique opportunity to establish the benefits of additional supplementation where it would not ordinarily be used in this cohort of patients.

Traditionally, vitamin D supplements are not prescribed if the serum 25 (OH) vitamin D level is around 70nmol/L as values above this are considered satisfactory yet there is no evidence to support this. Therefore the results of this ancillary study in conjunction with the ongoing clinical trial have the potential to modify current guidelines for vitamin D prescribing in patients with CKD with a view to significantly reducing the prevalence of cardiovascular morbidity and mortality in this patient group.

While most of the study schedule and procedures remain unaltered, the differences for this second study arm are detailed below.

**Investigational agent:**

This remains unchanged. We will use study medication for the original clinical trial which has previously been validated for use with an expiry date of August 31^st^ 2010.

**Old text**

## 1.5 Dose Rationale and Risk/Benefits

A dose regimen of ergocalciferol 50,000 IU weekly for 12 weeks and then monthly for 3 months has been shown to be effective at both raising serum vitamin D levels and lowering PTH levels in a similar cohort which is the precise effect we hope to achieve in our study group (28). No patient developed the most common side effect of hypercalcaemia and no other adverse effects were reported. A further study (29) of weekly ergocalciferol (50,000 IU/week for 24 weeks) was also shown to raise serum vitamin D levels with no adverse events reported. We have chosen the dose regimen in the K/DOQI guidelines for the replacement of vitamin D in patients with CKD. This consists of 50,000IU of ergocalciferol weekly for 1 month and then monthly for 5 months. As in the studies above, we anticipate a rise in serum vitamin D levels to therapeutic but well below toxic levels.

**New text**

**Dose rationale and risk/benefits**

A dose regimen of 50,000 IU ergocalciferol weekly for 1 month followed by 2 monthly doses of 50,000 IU ergocalciferol will be used. This will shorten the total study duration by 3 months (see study procedures). We have reduced the total dose to minimise the risk of hypercalcaemia in patients who may have a higher mean level of serum vitamin D. Patients will be monitored monthly and the investigators anticipate the risk of hypercalcaemia will be very low. Given that most patients with CKD are hypocalcaemic, we do not anticipate that hypercalcaemia as a result of vitamin D therapy is likely. To date in the existing clinical trial, there have been no laboratory confirmed cases of hypercalcaemia (serum calcium > 2.6 mmol/L).

**Study endpoints**

Cardiac MRI’s, echocardiograms, ECG’s and carotid ultrasound measurements will not be undertaken since there is likely to be very little significant change in these parameters over a three month study duration. The main focus of this study is the microcirculatory assessments which remain unchanged.

The inclusion criteria will be amended to include patients with a vitamin D level of <70nmol/L

**Old text**

Inclusion criteria

- eGFR between 15 and 60 ml/min/1.73m2
- Serum 25 (OH) vitamin D levels <40nmol/L as measured within the last 2 months
- No evidence of diabetes mellitus (fasting blood sugar <7.1, not taking any diabetic medication)
- Not receiving haemo or peritoneal dialysis
- No dialysis therapy within the last 3 months
- Age > 18 years and < 80 years
- Patient agrees not use any medications (prescribed or over-the-counter including herbal remedies) judged to be clinically significant by the Principal Investigator during the course of the study.
- Able to understand and sign the written Informed Consent Form.
- Able and willing to follow the Protocol requirements.

**New text**

Inclusion criteria

- eGFR between 15 and 60 ml/min/1.73m2
- Serum 25 (OH) vitamin D levels <70nmol/L as measured within the last 2 months
- No evidence of diabetes mellitus (fasting blood sugar <7.1, not taking any diabetic medication)
- Not receiving haemo or peritoneal dialysis
- No dialysis therapy within the last 3 months
- Age > 18 years and < 80 years
- Patient agrees not use any medications (prescribed or over-the-counter including herbal remedies) judged to be clinically significant by the Principal Investigator during the course of the study.
- Able to understand and sign the written Informed Consent Form.
- Able and willing to follow the Protocol requirements.

**The exclusion criteria remain unchanged**

**Study drug**

The existing study medication will be used for the additional study arm. Pharmacy staff at the Royal London Hospital will dispense 6 (six) capsules of study medication (either 50,000IU ergocalciferol or a matching placebo) per patient as per section 5 of the previous study protocol. Each container of study medication currently holds nine (9) capsules of study medication. Thus, three (3) capsules will be removed and destroyed from existing stock by the Royal London Pharmacy staff before being dispensed to study patients. The label on the current medication indicates there are 9 capsules per bottle. The label will be changed to reflect that only 6 capsules will be dispensed for the new study participants. This will be undertake by Mawdsley Brooks UK.

Highlighted text will be changed from “9” to “6” capsules and from “five” to “two”

# Old text

## Treatment Regimen

The replacement regimen is based on current guidelines for vitamin D replacement in CKD (33)

After randomization at visit 3, patients will take 1 capsule of study drug (either placebo or active ergocalciferol) weekly for four weeks and then monthly for the following 5 months (9 doses in total).

Study visit 3: After randomization, 9 capsules of study medication will be dispensed at this visit and the patient will be observed swallowing the first dose of medication. . Patients will be told to bring back the study medicine at the next and all subsequent visits.

Study visit 4: the number of capsules remaining will be counted and the patient will be observed swallowing the medication.

Study visit 5: the number of capsules remaining will be counted and the patient will be observed swallowing the medication.

Study visit 6: the number of capsules remaining will be counted and the patient will be observed swallowing the medication.

Study visit 7: the number of capsules remaining will be counted and the patient will be observed swallowing the medication.

Study visit 8: the number of capsules remaining will be counted and the patient will be observed swallowing the medication. This will be the last dose of study medication.

Study visit 9 (last visit): no medication will be taken.

No further study drug will be dispensed after visit 3 unless the patient has lost or mislaid the medication and has an insufficient supply to complete the trial

**New text**

## Treatment Regimen

After randomization at visit 3, patients will take 1 capsule of study drug (either placebo or active ergocalciferol) weekly for four weeks and then monthly for the following 2 months (6 doses in total).

Study visit 3: After randomization, 6 capsules of study medication will be dispensed at this visit and the patient will be observed swallowing the first dose of medication. . Patients will be told to bring back the study medicine at the next and all subsequent visits.

Study visit 4: the number of capsules remaining will be counted and the patient will be observed swallowing the medication.

Study visit 5: the number of capsules remaining will be counted and the patient will be observed swallowing the medication.

Study visit 6 (last visit): no medication will be taken.

No further study drug will be dispensed after visit 3 unless the patient has lost or mislaid the medication and has an insufficient supply to complete the trial

# Study Procedures and Schedule of Assessments

The follow up period has been shortened to three months. This will reduce the risk of hypercalcaemia and allow the investigators to determine if three months vitamin D therapy is sufficient to replenish vitamin D stores and modulate microvascular function. The total study duration for the additional arm is 4 months.

The table below details each study time point and exactly what will occur.

**OLD TEXT**

| **Visit** | **Drug** | **Action** | **Action** | **Action** | **Action** | **Action** | **Action** | **Action** | **Action** | **Action** |
| --- | --- | --- | --- | --- | --- | --- | --- | --- | --- | --- |
| 0 weeks | Washout |  |  |  |  |  |  |  | Bloods | Examination |
| 2 weeks | n |  | Microcirc |  |  |  |  |  |  | Examination |
| 1 month (4 weeks) | y | ECG | Microcirc | SF 36 | Urine | MRI/echo | 24hr BP | USS neck | Bloods | Examination |
| 2 month | y |  | Microcirc |  | Urine |  |  |  | Bloods | Examination |
| 3 months | y |  |  |  |  |  |  |  | Bloods | Examination |
| 4 months | y |  | Microcirc |  | Urine |  |  |  | Bloods | Examination |
| 5 months | y |  |  |  |  |  |  |  | Bloods | Examination |
| 6 months | y |  |  |  |  |  |  |  | Bloods | Examination |
| 7 months | n | ECG | Microcirc | SF 36 | Urine | MRI/echo | 24hr BP | USS neck | Bloods | Examination |

**NEW TEXT**

| **Visit** | **Drug** | **Action** | **Action** | **Action** | **Action** | **Action** |
| --- | --- | --- | --- | --- | --- | --- |
| 0 weeks | Washout |  |  |  | Bloods | Examination |
| 2 weeks | n | Microcirc |  |  |  | Examination |
| 1 month (4 weeks) | y | Microcirc | SF 36 | Urine | Bloods | Examination |
| 2 month | y | Microcirc |  | Urine | Bloods | Examination |
| 3 months | y |  |  |  | Bloods | Examination |
| 4 months | n | Microcirc | SF 36 | Urine | Bloods | Examination |

Each Action is listed in section 7 of the previous study protocol.

**OLD TEXT**

**Drug** – at month 1 (week 4) patients will be randomized to and receive at that visit either placebo or ergocalciferol. Patients will receive all 9 capsules of study medication at this time. They will be required to take 1 dose weekly at home in the first month. Further study medication will be taken at subsequent clinic visits and patients will be observed swallowing their medication either by pharmacy staff or an investigator.

**NEW TEXT**

**Drug** – at month 1 (week 4) patients will be randomized to and receive at that visit either placebo or ergocalciferol. Patients will receive all 6 capsules of study medication at this time. They will be required to take 1 dose weekly at home in the first month. Further study medication will be taken at subsequent clinic visits and patients will be observed swallowing their medication either by pharmacy staff or an investigator.

**Other blood tests**

These tests will be taken at visits 3 and 6

1,25 OH vitamin D, Trop T (troponin T), renin, aldosterone

## Sample Size Determination

This is a pilot study for which there is no existing data to generate a reliable power calculation. Based on clinical experience, the investigators consider that 15 patients will be required in both the placebo and treatment arm to demonstrate a significant difference between the groups.

**All other aspects of the study protocol remain unchanged from version 1.2**

FINAL PAGE

INTENTIONALLY BLANK
