# Supplementary material for: Ergocalciferol and Microcirculatory Function in Chronic Kidney Disease and Concomitant Vitamin D Deficiency: An Exploratory, Double Blind, Randomised Controlled Trial
Source: PLoS One. 2014 Jul 9;9(7):e99461. doi: 10.1371/journal.pone.0099461 (PMC4090117; doi:10.1371/journal.pone.0099461)
Supplement: File S1 — Full methodology. Contains Table S1, full iontophoresis protocol. (DOCX) [file pone.0099461.s008.docx]

**SUPPORTING INFORMATION**

**Ergocalciferol and microcirculatory function in chronic kidney disease and concomitant vitamin D deficiency: an exploratory, double blind, randomised controlled trial**

**Dreyer et al.**

**Complete methods**

**Patient preparation**

Patients were instructed to wear loose clothing, avoid caffeine and nicotine and rested for 15 minutes in an environmentally controlled room before microvascular assessments. Clinical assessments were conducted in the following order to avoid any systemic effect of drugs delivered during iontophoresis: side stream dark field imaging of the sublingual microcirculation, AGE measurement, pulse wave velocity and iontophoresis.

**Practical Achievement of iontophoresis**

The skin of the volar aspect of the forearm was prepared by gentle wiping with an alcohol street which removes a thin layer of dead skin which could theoretically interfere with laser Doppler assessment. The skin was left exposed to room air to dry for 60 seconds after wiping to avoid any vasoactive effects as a result of the cooling effect of the alcohol rub.

Two iontophoresis chambers (Moor Instruments, UK) were adhered to the skin of the non dominant forearm. The proximal chamber was located approximately 3 cm from the lower elbow crease and chambers were separated by at least 5 cm to avoid the vasoactive response at one chamber being detected at the other chamber site. Both chambers were sited to avoid hair, scars, freckles and visible or palpable blood vessels in order to maximize the chance of the laser probes obtaining a true recording from dermal microvessels. The Doppler probes (Moor instruments, UK) were inserted into the centre of the ion chamber and approximately 100 µl per chamber of a 1% solution of Acetylcholine (ACh) (Novartis, UK) or Sodium Nitroprusside (SNP) (Mawdsley Brooks, UK) were introduced to the centre of the chamber via 2 holes in its upper surface. ACh was inserted at the anode and SNP at the cathode.

The laser probes were connected to a laser Doppler monitor (DRT4 – Moor instruments, UK) which is in turn connected to a current delivery device (MIC 1 – Moor instruments, UK). The hardware is supported and controlled by integrated software (laser Doppler perfusion monitor v1.2, Moor Instruments UK) which allows for simultaneous laser Doppler recording and delivery of the iontophoretic protocol via the MIC 1 device. The iontophoretic protocol used a low current protocol of sequential dose increases of ACh and SNP (Table S1) in order to avoid the galvanic effect of drug delivery itself on vasoactive changes in the microcirculation.(1) Baseline flux was measured in study subjects for 60 s before iontophoresis was commenced. Relative change from baseline flux, measured as a percentage, is the primary outcome data used for analysis.(2) An example of the iontophoretic output is shown in Figure S1. Differences between treatment groups are compared using the Student t-test. A p value of <0.05 is considered statistically significant.

**Pulse wave velocity measurement**

Patients were prepared as for iontophoresis and in addition were reclined to approximately 15 degrees from horizontal. This technique utilised the Vicorder device (Skidmore Medical). For the measurement of the aortic segment pulse wave velocity, a cuff was placed around the neck over the carotid artery and a second cuff is placed around the upper thigh. These cuffs were connected to the control box which is in turn connected to a computer running the Vicorder software. With the patient recumbent, the distance between the suprasternal notch and the upper border of the thigh cuff is recorded, reflecting the approximate distance of the arterial segment being measured.

Both cuffs were inflated simultaneously to approximately 70 mmHg. The cuffs remained inflated for between 3 and 5 beats (or pulse transmissions). Pulse wave speed (or transit time) was recorded and the PWV was generated by dividing the distance between the suprasternal notch and the thigh cuff in cm by the transit time (time taken in m/s for the pulse wave to travel between the 2 sensor cuffs). Readings were taken from the left and right thigh and the mean value is presented as aortic pulse wave velocity.

**Measurement of tissue advanced glycation end products.**

This technique utilised the Diagnoptics AGE reader (Netherlands). This device provides a non-invasive measure of advanced glycation end products (AGE) in tissues exposed to the illumination field of the device.

The AGE-Reader consists of a small box, containing an excitation light source, emitting light with wavelengths of 300–420 nm (peak ~370 nm). Patients place their forearm over this box. The mean of three separate readings of reflectance is converted to a measure of skin autofluoresence (AF). An area of the volar aspect of the forearm free from scars or tattoos was selected for measurement. The skin was gently cleaned with an alcohol wipe. To correct for differences in light absorption, skin AF is calculated by dividing the amount of emitted light intensity between 420 and 600 nm by the amount of excitation light intensity between 300 and 420 nm, expressed as arbitrary units.

**Side stream, dark field (SDF) imaging of the sublingual microcirculation.**

This technique utilised the Microscan probe (Microscan Medical, The Netherlands). A small camera (0.7cm lens tip) with a replaceable sterile cap was placed under the tongue. Three separate video images of 60 s duration are collected during one reading. Each image is taken from a separate area of the sublingual capillary bed by gentle repositioning of the camera tip.

An analysis of the moving cells in the images permits the quantitative measurement of red blood cell flow in the capillaries. Additionally, morphological characteristics of the microcirculation, such as functional capillary density and micro-vessel morphology, can be measured using reflectance avoidance imaging. SDF images were scored and interpreted according to standard consensus guidelines.(3)

**Cardiac magnetic resonance imaging (cMRI)**

cMRI was performed using a Philips Achieva CV 1.5T. Left ventricular mass (LVM) was measured from the steady state free precession contiguous short axis cine stack (8mm slice thickness and 2mm interslice gap, with whole LV coverage) using Philips MR WorkSpace software. Left ventricular mass and left ventricular mass indices (LV mass normalized to body surface area) were measured directly by this technique. Study patients underwent cMRI imaging for LVMI at baseline and 6 months.

**Cell experiments**

Human Aortic Endothelial Cells (HAEC) (Promocell) were cultured and passaged on 75 cm^2^ cell culture flasks using Endothelial Cell Growth Medium MV (Promocell)

When the desired confluence was obtained, experimental compounds were added to the cultured cells in the cell media. Vitamin D in the form of ergocalciferol (Sigma) was diluted in ethanol to the appropriate concentration. Two control experiments were conducted synchronously by the addition of media alone and media with ethanol, each added at the same volume as ergocalciferol to the cell media (10 µL for final dilutions).

Two experimental arms were conducted using either high (120 ng/dl ergocalciferol) or low (12 ng/dl ergocalciferol) concentrations of vitamin D compounds. Ergocalciferol was used since this reflects established guidelines for vitamin D replacement in patients with CKD.(4)

Cells were incubated with experimental compounds for 0 and 24 h. At the relevant time point, the cells were scraped from the culture wells and the resulting cell suspension was aspirated and centrifuged at 1000 *g* for 5 min in a pre-cooled centrifuge at 4^0^C. The supernatant was aspirated and stored at -20^0^C and the cell pellet immediately frozen at -80^0^ C.

### Real time polymerase chain reaction (RT-PCR) for eNOS

To establish the fold increase of eNOS expression compared to a control of β actin gene expression, real time polymerase chain reaction (RT-PCR) on cell lysates was performed. Messenger RNA (mRNA) was extracted from cell lysates using commercially available kits (QIAGEN RNeasy mini kit, UK) according to the manufacturer’s instructions.

0.75 µg of mRNA was added to 1.2 µL of Oligo DT primer (Invitrogen (12-18, 0.5 μg/μL)) and 1.2 µL of 100 nM 2'-deoxynucleoside 5'-triphosphate (dNTP) Genetic grade water (Purelab Ultra, ELGA) was added to a total volume of 14 µL. This mixture was heated to 65^0^C for 5 min and then immediately chilled on ice. The contents were then briefly centrifuged before the addition of 4.7 µL of 5x first standard buffer (Invitrogen), 2.3µL of 0.1M dithiothreitol (DTT (Invitrogen)) and 1 µL superscript reverse transcriptase (Invitrogen). The mixture was briefly agitated before incubation at 42^0^C for 50 min and then inactivation at 70^0^C for 15 min.

2 µL of the resulting cDNA mixture was added to 0.4 µL of gene mix (either eNOS or β actin Taqman gene expression assay: eNOS assay ID: Hs01574659_m1, β actin assay ID: Hs99999903_m1), 4 µL of Master mix (Thermo Scientific) and 3.6 µL of dH_2_0 to a total volume of 10 µL. The mixture was centrifuged briefly before PCR was performed on the Applied Biosystems 7900HT Fast Real-Time PCR System. The PCR conditions were one 2 min cycle (50 °C), denaturation for 10 minutes (95 °C) followed by 40 cycles of denaturation (95 °C for 15 s). Annealing and extension were performed as a single step (60 °C for 1 min). Data were analyzed with ABI 7900HT Prism sequence detector software (SDS Version 2.3, Applied Biosystems) using the ΔΔCT method to determine differential gene expression between experimental compounds.

### Measurement of cell supernatant nitrite levels

Nitrite levels in supernatant from HAEC cultures was analysed using a chemiluminescent technique.(5) HAEC supernatant was filtered through a washed microcon YM-3 filter at 4°C, 14,000 g for 90 min and the filtrate snap frozen and stored at -80°C until use. An NO analyser (NOA 280A, Sievers) was used to measure NO based on the gas-phase chemiluminescent reaction between NO and ozone (O₃). Statistical analysis was performed using GraphPad Prism software (version 5). Differences in nitrite levels were assessed using the Student t-test.

**Sub group analysis for the primary outcome**

The iontophoretic response was assessed separately in patients with hypertension (ergocalciferol n=5, placebo n=7) and glomerulonephritis (ergocalciferol n=8, placebo n=5) using the same statistical approach as described in the methods section of the main manuscript.

In patients with hypertension comparing the effect of ergocalciferol and placebo, there was no significant difference between percentage increase from baseline flux after either iontophoresis of ACh (2 way ANOVA with repeated measures p=0.83, Bonferroni post tests at all time points p>0.05) or SNP (2 way ANOVA with repeated measures p=0.73, Bonferroni post tests at all time points p>0.05) (see Figure S2 and S3).

The findings were similar for patients with glomerulonephritis comparing the effect of ergocalciferol and placebo after iontophoresis of ACh (2 way ANOVA with repeated measures p=0.77, Bonferroni post tests at all time points p>0.05) and SNP (2 way ANOVA with repeated measures p=0.67, Bonferroni post tests at all time points p>0.05) (see Figure S4 and S5). The statistical significance was not changed when mixed effects models were used in either the hypertension or glomerulonephritis sub groups. The absence of statistically significant differences in the placebo and ergocalciferol treated patients in the sub-group analysis may be due to the small numbers of subjects in each of the hypertension and glomerulonephritis groups.

Table S1 Iontophoretic protocol for patients with CKD and healthy volunteers.

| **Epoch** | **Current (μA)** | **ACh** | **SNP** |
| --- | --- | --- | --- |
| **1** | 0 (baseline) | 60 s | 60 s |
| **2** | 10 | 30 s | 30 s |
| **3** | 0 | 60 s | 60 s |
| **4** | 20 | 30 s | 30 s |
| **5** | 0 | 60 s | 60 s |
| **6** | 35 | 30 s | 30 s |
| **7** | 0 | 60 s | 60 s |
| **8** | 50 | 30 s | 30 s |
| **9** | 0 | 60 s | 60 s |
| **10** | 75 | 30 s | 30 s |
| **11** | 0 | 60 s washout | 60 s washout |

**References for supplemental data**

1. Droog EJ, Henricson J, Nilsson GE, et al. A protocol for iontophoresis of acetylcholine and sodium nitroprusside that minimises nonspecific vasodilatory effects. *Microvasc Res*. 2004; **67**: 197-202.

2. Cupisti A, Rossi M, Placidi S, et al. Responses of the skin microcirculation to acetylcholine and to sodium nitroprusside in chronic uremic patients. *Int J Clin Lab Res*. 2000; **30**: 157-62.

3. De Backer D, Hollenberg S, Boerma C, et al. How to evaluate the microcirculation: report of a round table conference. *Crit Care*. 2007; **11**: R101.

4. K/DOQI Clinical Practice Guidelines for Bone Metabolism and Disease in Chronic Kidney Disease. *Am J Kidney Dis*. 2003; **42**: S1-S201.

5. Ignarro LJ, Fukuto JM, Griscavage JM, et al. Oxidation of nitric oxide in aqueous solution to nitrite but not nitrate: comparison with enzymatically formed nitric oxide from L-arginine. *Proc Natl Acad Sci U S A*. 1993; **90**: 8103-7.
